# Molecular Evolution of Cytochrome *bd* Oxidases across Proteobacterial Genomes

**DOI:** 10.1093/gbe/evv032

**Published:** 2015-02-16

**Authors:** Mauro Degli Esposti, Tania Rosas-Pérez, Luis Eduardo Servín-Garcidueñas, Luis Manuel Bolaños, Monica Rosenblueth, Esperanza Martínez-Romero

**Affiliations:** ^1^Italian Institute of Technology, Genoa, Italy; ^2^Center of Genomic Sciences, UNAM University, Cuernavaca, Mexico

**Keywords:** bioenergetics, cytochrome *bd* oxidase, proteobacteria, bacterial evolution

## Abstract

This work is aimed to resolve the complex molecular evolution of cytochrome *bd* ubiquinol oxidase, a nearly ubiquitous bacterial enzyme that is involved in redox balance and bioenergetics. Previous studies have created an unclear picture of *bd* oxidases phylogenesis without considering the existence of diverse types of *bd* oxidases. Integrated approaches of genomic and protein analysis focused on proteobacteria have generated a molecular classification of diverse types of *bd* oxidases, which produces a new scenario for interpreting their evolution. A duplication of the original gene cluster of *bd* oxidase might have occurred in the ancestors of extant α-proteobacteria of the Rhodospirillales order, such as *Acidocella*, from which the *bd-I* type of the oxidase might have diffused to other proteobacterial lineages. In contrast, the Cyanide-Insensitive Oxidase type may have differentiated into recognizable subtypes after another gene cluster duplication. These subtypes are widespread in the genomes of α-, β-, and γ-proteobacteria, with occasional instances of lateral gene transfer. In resolving the evolutionary pattern of proteobacterial *bd* oxidases, this work sheds new light on the basal taxa of α-proteobacteria from which the γ-proteobacterial lineage probably emerged.

## Introduction

Since its original introduction, the *phylum* of proteobacteria occupies a pivotal position in the classification of Eubacteria (Woese 1987; Stackebrandt et al. 1988; Gupta and Mok 2007; Williams et al. 2007). Proteobacteria are divided into five classes forming two major groups: The early diverging group predominantly contains anaerobic organisms of the δ and ε classes, and the other includes predominantly aerobic and facultatively anaerobic organisms classified under the α, β, and γ classes. The γ class includes Enterobacteria. The class of α-proteobacteria appears to be the most diversified in phylogenetic terms; seemingly it emerged before the separation of the β and γ lineages—at the time in which ambient oxygen levels dramatically increased on the planet (Battistuzzi et al. 2004)—and then originated the protomitochondria, from which the mitochondrial organelle of eukaryotic cells evolved (Gray et al. 2001; Williams et al. 2007). The interest on the origin of protomitochondria has promoted evolutionary studies on α-proteobacteria (Brindefalk et al. 2011; Müller et al. 2012; Ferla et al. 2013; Degli Esposti et al. 2014; Amrine et al. 2014; Le et al. 2014).

Currently, α-proteobacteria are subdivided in recognized orders such as Rhodospirillales (Gupta and Mok 2007; Williams et al. 2007; Le et al. 2014) and a growing group of “unclassified” organisms, often living in marine habitats and possessing streamlined genomes with unclear phylogentic positions (Wagner-Döbler and Biebl 2006—*Roseobacter*; Davidov et al. 2006—*Micavibrio*; Foesel et al. 2007—*Geminicoccus*; Albuquerque et al. 2008—*Elioraea*; Bazylinski et al. 2013—*Magnetococcus*; Jiang et al. 2014—*Defluviimonas*; Brindefalk et al. 2011; Viklund et al. 2012; Grote et al. 2012; Amrine et al. 2014). Despite some convergence in locating the Rickettsiales, a group containing only obligate endocellular parasites, at the base of the phylogenetic trees of α-proteobacteria (e.g., Williams et al. 2007), the branching order remains fundamentally unresolved, especially when mitochondria are included in the analysis (Ferla et al. 2013). Recently, the origin of protomitochondria has been investigated with the novel approach of following the progressive loss of the bioenergetic systems that are present in the genomes of extant α-proteobacteria (Degli Esposti et al. 2014). The loss of the cytochrome *bd* oxidase, also known as *bd*-type ubiquinol oxidase, appears to be the final step in the most likely pattern of protomitochondria evolution as well as in the transition from free living to mutualistic symbionts of extant bacteria (Degli Esposti 2014).

Formed by two conserved transmembrane subunits binding only hems as redox groups, cytochrome *bd* oxidase is present in most bacterial *phyla* and in some Archaea, but not in mitochondria or other eukaryotic organelles (Borisov et al. 2011). The term *bd* oxidase encompasses two similar types of bioenergy-producing quinol oxidoreductases: The *bd-*I type present in *Escherichia coli* (Osborne and Gennis 1999; Borisov et al. 2011) and the Cyanide-Insensitive Oxidase (CIO) type (Cunningham et al. 1997; Mogi 2009). The latter is recognized from the structure of its catalytic subunit I, which lacks the large extension in the so-called “Q-loop” protruding at the periplasmic side of the membrane (Osborne and Gennis 1999; Sakamoto et al. 1999; Borisov et al. 2011) and consequently is significantly shorter than its homologues of the *bd-*I type. In contrast, the second *bd* oxidase of *E. coli* and other Enterobacteria, *bd*-II, is rather similar to the *bd*-I type but clearly different from the CIO type (Borisov et al. 2011). Despite the apparent lack of overall diversity (Borisov et al. 2011), the absence or presence of the long Q loop extension is generally sufficient to distinguish the *bd-*I(I) type from the CIO type of proteobacterial *bd* oxidases (Degli Esposti et al. 2014).

The phylogenetic value of the Q loop extension is blurred by its unclear origin. Firmicutes (Gram-positive bacteria such as *Bacillus*) possess multiple short versions of the catalytic subunits which are clearly different from each other, even if they all lack long Q loop extensions (Winstedt et al. 1998; Sakamoto et al. 1999; Winstedt and von Wachenfeldt 2000). In contrast, subunit I of some Archean *bd* oxidases has extrinsic extensions that do not correspond to those seen in proteobacterial *bd-*I(I) type. Consequently, the resulting picture of *bd* oxidase phylogeny is confusing and looks inconsistent with the current classification of bacteria (Brochier-Armanet et al. 2009), a widely accepted concept which has suggested extensive events of lateral gene transfer (LGT) across distant taxa (Hao and Golding 2006; Brochier-Armanet et al. 2009; Borisov et al. 2011; Chouaia et al. 2014). However, the phylogenetic studies reported to date have ignored the existence of two basic types of *bd* oxidases, which are differentially distributed in bacterial genomes (Degli Esposti et al. 2014). Herein we present a classification of the genomic organization of *bd* oxidases from proteobacteria, which are by far the most studied. The classification produces a new scenario for the evolution of the enzyme that bears relevance to the early differentiation of α- and γ-proteobacteria. An early duplication of the ancestral gene cluster of *bd* oxidase can be reconstructed from the genomic organization of the *bd* oxidases that are present in α-proteobacteria belonging to the Rhodospirillales order, such as *Acidocella*. The *bd*-I type with its characteristic long Q loop extension originated from one duplicate set of genes that remained relatively conserved in spreading across proteobacterial lineages. Conversely, the other set of duplicated genes might have been lost whereas ancestral CIO-like proteins were separately inherited from Firmicutes, producing two different subtypes of CIO *bd* oxidase. These subtypes are differentially present in α-, β-, and γ-proteobacterial lineages, sometimes in multiple copies within a single genome due to additional duplications. Hence, this work presents a possible resolution of the molecular evolution of *bd* oxidases in proteobacteria.

## Materials and Methods

The approaches followed in this work are partially derived from those recently introduced for studying the evolution of mitochondria (Degli Esposti et al. 2014). The program Domain Enhanced Lookup Time Accelerated BLAST, DELTABLAST (Boratyn et al. 2012), has been extensively used to identify genes and their products using the entire NR (Nonredundant) database, or selection thereof from the National Center for Biotechnology Information (NCBI). Alignments of protein sequences have been refined manually (Tron et al. 1991) after initial drafts obtained with the COBALT resource of the DELTABLAST program (Boratyn et al. 2012) had been integrated with hydropathy analysis conducted with the program WHAT (Web-based Hydropathy, Amphipathicity and Topology; http://saier-144-21.ucsd.edu/barwhat.html, last accessed 4 January 2015; Cuff et al. 1998). Phylogenetic trees of manually curated alignments were subsequently obtained with the program PhyML 3.0 (Guindon et al. 2010). When the match between DELTABLAST-generated trees and those obtained with PhyML was poor, phylogenetic trees were generated after refinement of the sequence alignments with Gblocks (Talavera and Castresana 2007). Bioinformatics data have been further integrated with available biochemical and microbiological information (see Degli Esposti et al. 2014 and Borisov et al. 2011, and references therein). Operon classification and nomenclature were taken from Price et al. (2006) as described earlier (Degli Esposti et al. 2014).

Maximum-likelihood (ML) trees were produced with 100 bootstrap replicates using also the RAxML program (Stamatakis et al. 2008) and the amino acid substitution matrix BLOSUM62, which is the default matrix in DELTABLAST searches (Boratyn et al. 2012). Extended majority rule consensus tree of the 100 replicates was generated as described earlier (Stamatakis et al. 2008). For short hydrophobic proteins such as *cydX*, we have initially implemented manual alignments of sequences from over 50 phylogenetically different taxa that were guided by the estimated length and position of the transmembrane segment(s) predicted with either the WHAT online program or in house algorithms of hydropathy (Degli Esposti et al. 2014). In these alignments, we minimized the number and size of gaps required to optimize sequence identity within predicted transmembrane segments, favoring single residue gaps or those maintaining helical periodicity, verified also with the profile of amphypaticity generated by the WHAT program (Cuff et al. 1998). The original protein sequences were then retrieved from NCBI resources and aligned with MUSCLE, which failed to produce compact alignments as any other program tested, for instance ClustalW. These alignments were subsequently modified to precisely match those manually implemented and then run with the program PhyML to generate phylogenetic ML trees using the LG model for amino acid substitutions (Le and Gascuel 2008). We found this model to be superior to others such as the Whole Genome Alignment (WGA) and JTT (Jones, Taylor, and Thorton) model, especially for basal taxa and the match in tree topology with other subunits of *bd* oxidases. Indeed, the LG model gives more realistic substitution frequencies to key aromatic residues such as Trp that are concentrated at the beginning of the transmembrane segments in small subunits of bacterial oxidases, such as *COX*4 (Degli Esposti et al. 2014). The same procedure was used to evaluate phylogenetic features in concatenated functional domains of the catalytic subunit I of *bd* oxidases.

Conversely, the PHYLOPHLAN pipeline (Segata et al. 2013) was employed for the phylogenomic approach of building phylogenetic trees with at least 30 bacterial genomes. The program uses the predicted proteomes recovered from sequenced genomes and tries to locate 400 conserved proteins; then it performs individual protein alignments using MUSCLE with the protein sets recovered from the input genomes, subsequently concatenating the most discriminative positions in each protein alignment into a single sequence (Segata et al. 2013). After refining such concatenated alignments for approximately 400 conserved proteins, the program finally reconstructs phylogenetic trees using FastTree and an ML approach, defining local support values with 1,000 resamples. The tree outputs in newick format were visualized and edited with the TreeDyn 198.3 web-based program and the TreeGraph 2.1 program (Stover and Muller 2010).

Primers specific for *Acidocella cydA* were designed with OLIGO 6.0 software on the basis of available nucleotide sequences that were aligned in regions of high protein conservation using the program DAMBE (Xia 2013).

## Results and Discussion

### Premise

**Fig. 1.—:**
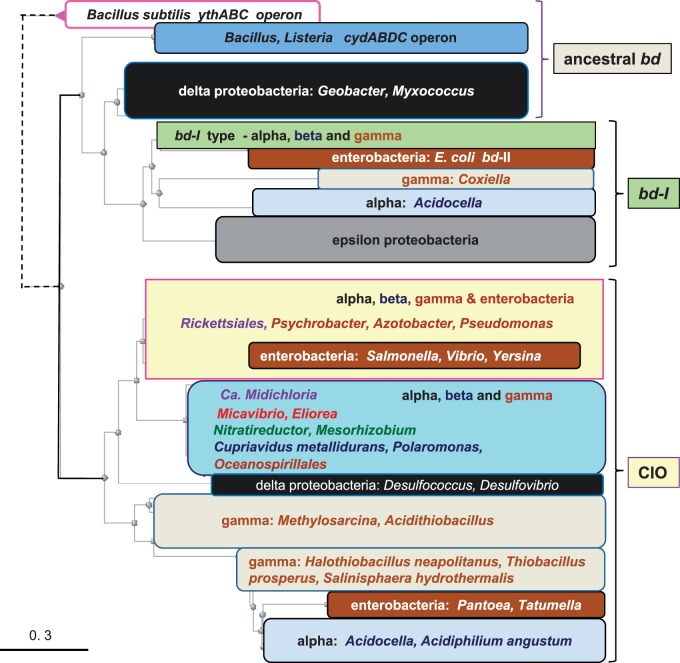
Representative phylogenetic tree of all *bd* oxidases of proteobacteria. DELTABLAST (Boratyn et al. 2012) searches were run against 10,000 sequences of the latest version of the NR database (accessed on October 18, 2014) using the 480 residues long *cydA* protein of *Acidocella facilis* (accession: WP_026438987) as a query, to produce a robust topology of the *bd* oxidases from all proteobacteria and a selection of *Bacillus* species harboring both *cydA* and *ythA* proteins (Winstedt et al. 1998; Winstedt and von Wachenfeldt 2000). Subsequently, the distance tree that best matched this topology was derived from iterative DELTABLAST searches over a selection of 1,000 sequences that contained all taxa representing the various classes of proteobacteria, including the Enterobacteria distributing along different major nodes in the original tree. The color-coded blocks of taxa overlay one of such a Neighbor Joining (NJ) tree that clearly shows the separation between the *bd*-I subtype (top) from the CIO type (bottom) of all proteobacterial *bd* oxidases. The distance of the divergent *ythA* sequences has been shortened (dashed lines on the top left) to expand the view on the major nodes of the tree.

**Fig. 2.—:**
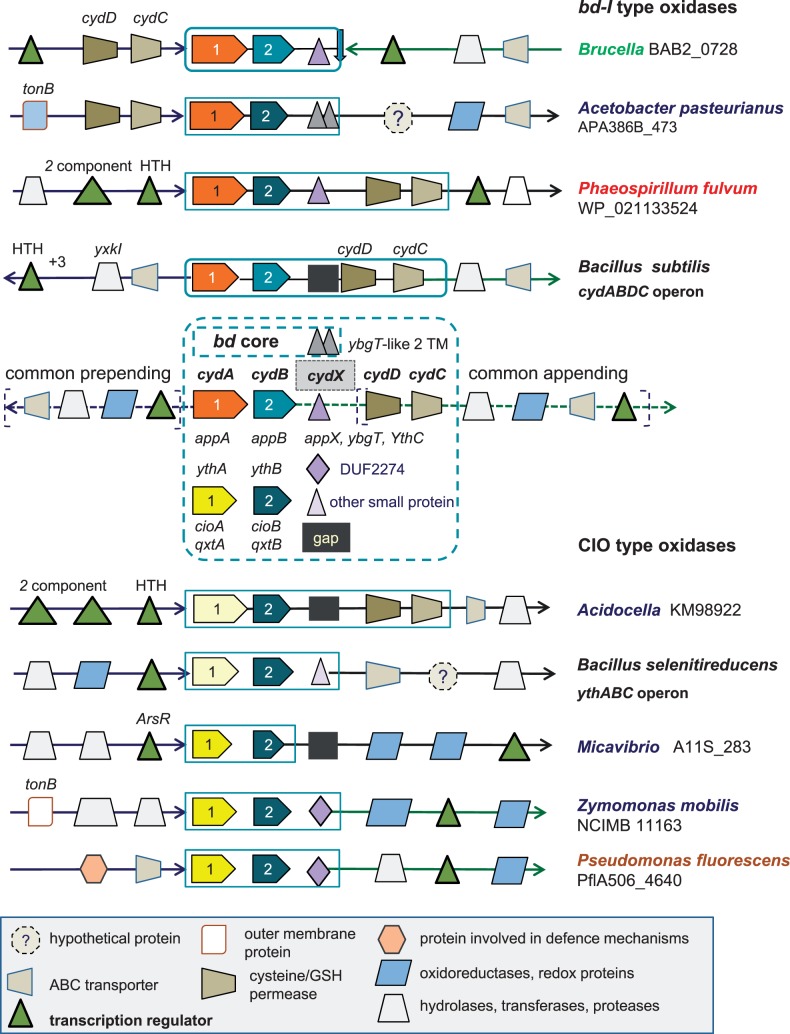
Graphical representation of the characteristics of the gene clusters for *bd* oxidases. The basic structure of the gene clusters for *bd* oxidases conforms to those of *cydABDC* (top) and *ythABC* (bottom) operons found in *Bacillus* organisms (Winstedt et al. 1998; Winstedt and von Wachenfeldt 2000). The gene accession for *cydA* is reported for several taxa. Diverse graphical symbols indicate functionally different proteins, as illustrated in the legend at the bottom of the figure. The various names for gene annotation of the core subunits of *bd* oxidases are listed within the central dashed square. Structurally different forms of the small hydrophobic subunit *cydX* are represented as follows: Two adjacent triangles, two transmembrane segment-proteins of acetic acid and other bacteria; a triangle, the single transmembrane *cydX* proteins of *bd*-I type oxidases; a rhombic, the single transmembrane DUF2474 proteins. A black square indicates the apparent absence of this subunit (gap) in the gene cluster.

**Fig. 3.—:**
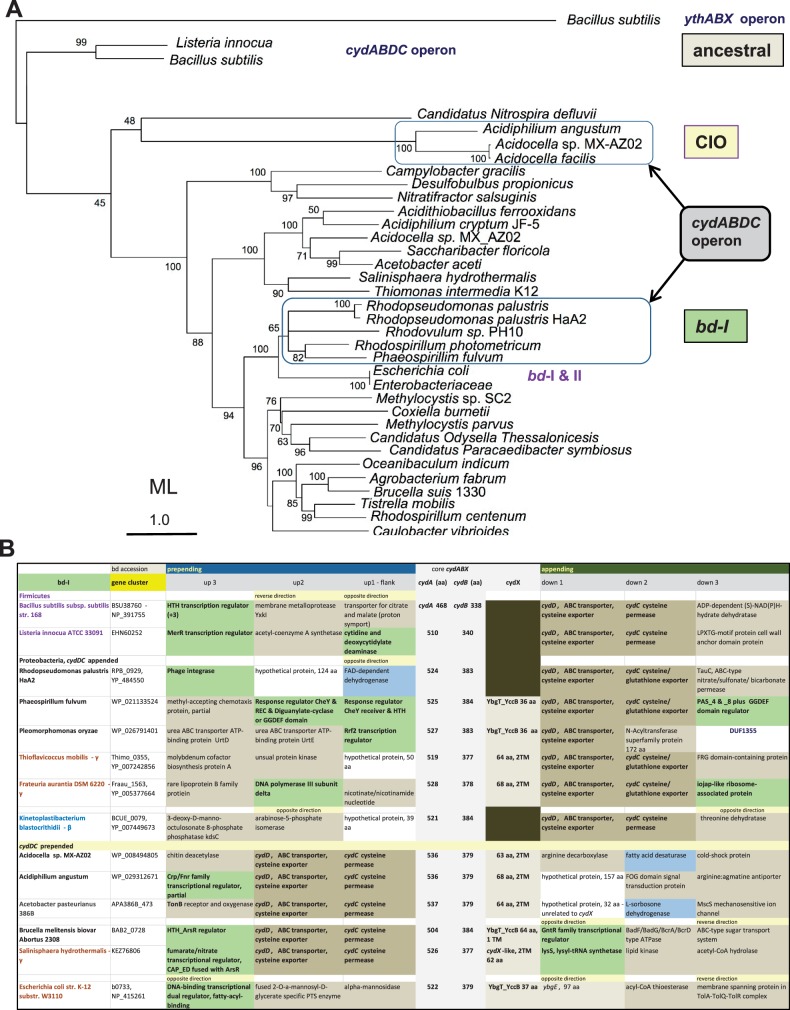
Phylogenetic tree and gene cluster of representative *cydA* proteins of *bd*-I type oxidases. (*A*) The phylogenetic ML tree was generated with the program RAxML using as a guide the NJ tree that was obtained with a wide DELTABLAST search using the *cydA* protein of the δ-proteobacterium, *D. propionicus* (accession: YP_004194034), as a query over a selection of taxa representing all major groups containing *bd*-I type oxidases (cf. fig. 1). The top branch containing sequences of CIO type oxidases is represented only by two species of *Acidocella* and *A. angustum* which retain the original *cydABDC* operon of *Bacillus*. The group of *bd*-I sequences that belong to the same operon is also indicated. (*B*) The table contains basic information for the gene clusters for *bd*-I type oxidases of the various taxa that are indicated on the left, with three prepended and three appended positions around the core genes of the oxidases. Color coding for different genes is as follows (cf. fig. 2): Dark brown, ABC-type transporters such as the cysteine exporter *cydC*; pale brown, enzyme or protein involved in metabolic reactions (not redox); pale blue, enzyme or protein involved in redox reactions; bright green, protein involved in DNA or RNA binding and regulation; DUF number in dark blue font, protein belonging to the specified family of DUF. Note that in the first row of Firmicutes the prepended HelixTurnHelix transcription regulator lies three positions upstream that indicated by the symbol +3. All other entries occupy the indicated position in the table, which can be obtained upon request.

**Fig. 4.—:**
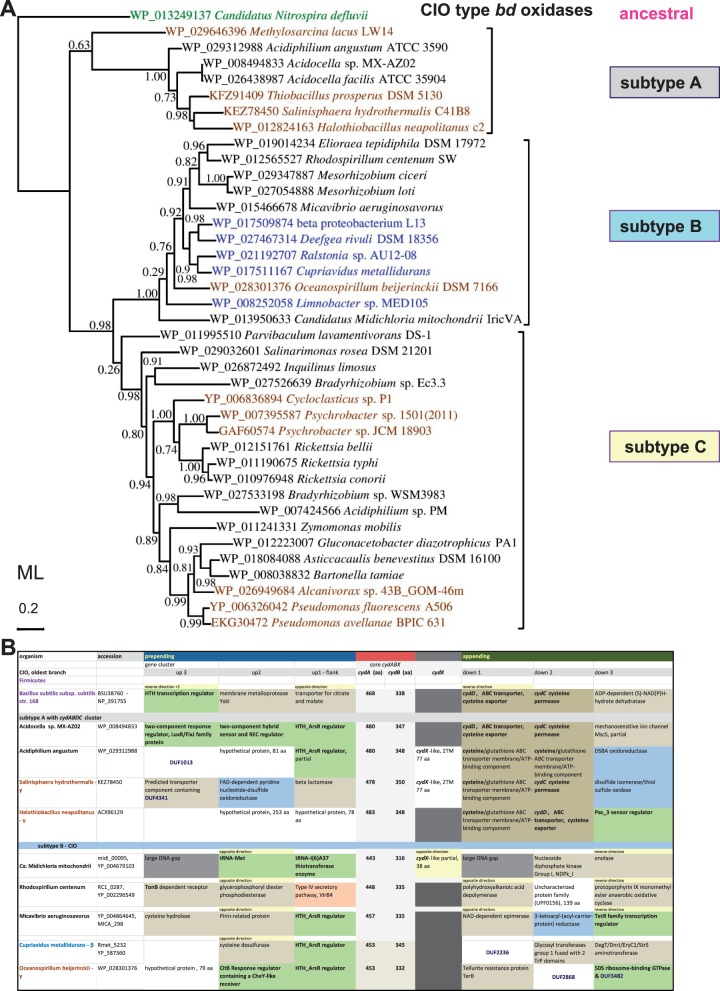
Phylogenetic tree and gene clusters of representative *cydA* proteins of CIO type oxidases. (*A*) The phylogenetic ML tree of *cydA* proteins (subunit I of *bd* oxidase) was obtained with the Program PhyML using as a guide NJ trees generated by a wide DELTABLAST search (cf. supplementary fig. S1, Supplementary Material online). The tree was refined by using Gblocks (Talavera and Castresana 2007) for improving the alignment of selected sequences, which are shown with their specific accession on the diagram given the presence of multiple *cydA* proteins in some taxa (see text). The classification of the proteins in subtypes A, B, and C follows that emerged from the enlarged analysis shown in supplementary figure S1, Supplementary Material online. The horizontal bar on the left indicates the fraction of amino acid changes estimated per site for a unit of branch length. Shimodaira–Hasegawa (SH)-like local support values are shown adjacent to nodes. (*B*) The table contains basic information for the gene clusters of CIO type from taxa that are representative for subtypes A and B, following the same color coding as in figure 3*B*.

**Fig. 5.—:**
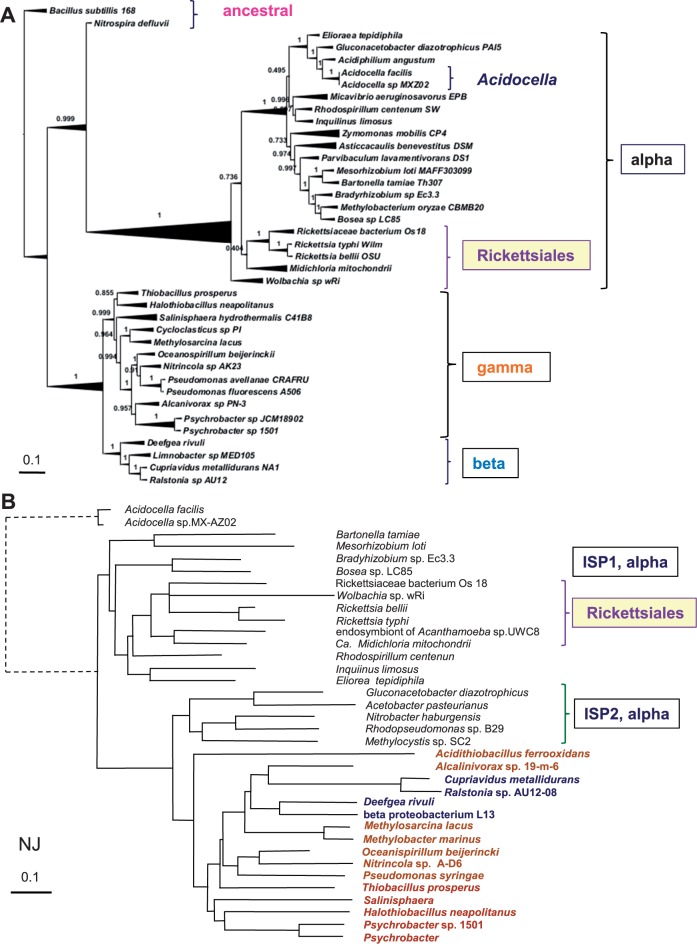
Phylogenomics of taxa used in *bd* oxidase trees versus the phylogenetic tree of ISP. (*A*) The tree was constructed with the phylogenomic approach of Segata et al. (2013) for the taxa used in figures 3 and 4, as described in the Materials and Methods section. Note the position of *Nitrospira* close to the branch of α-proteobacteria. See supplementary figure S2, Supplementary Material online, for a comprehensive view of the phylogenesis of γ-proteobacteria. SH-like local support values are shown adjacent to nodes. (*B*) The NJ tree was derived from a wide DELTABLAST search using as a query the ISP from the γ-proteobacterium, *Halothiobacillus neapolitanus* (accession: ACX95974), over a selection of taxa including all α-proteobacteria possessing the ISP2 form of the protein that is similar to the ISP of γ-proteobacteria (cf. Degli Esposti et al. 2014). The distance of the divergent *Acidocella* sequences has been shortened (dashed lines on the left) to expand the view on the major nodes.

**Fig. 6.—:**
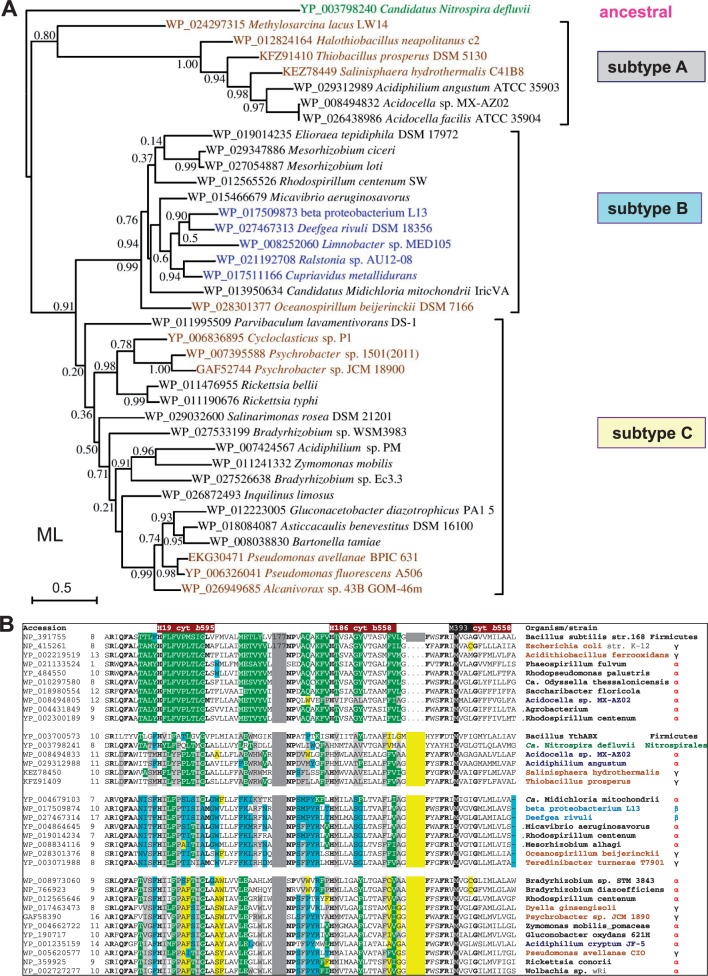
(*A*) Phylogenetic tree of representative *cydB* proteins of CIO type oxidases. The ML phylogenetic of tree *cydB* proteins (subunit II of *bd* oxidase) was built as in figure 4 *A* using as a guide the NJ tree obtained with a wide DELTABLAST search using as a query the 340 residues long *cydB* protein (subunit II) of *Ca. Nitrospira defluvii* (accession: YP_003798240) over a selection of taxa representing major groups of CIO oxidases, with the exception of δ-and Enterobacteria (see text). The tree was built on an improved alignment of the selected sequences (shown with their specific accession on the diagram as in fig. 4*A*) that was obtained using Gblocks (Talavera and Castresana 2007). The horizontal bar on the left indicates the fraction of amino acid changes estimated per site for a unit of branch length, whereas SH-like local support values are shown adjacent to nodes. Note the nearly complete match in topology with the tree obtained for the *cydA* protein (fig. 4*A*). However, topology differences with respect to the *cydA* tree of the same taxa (cf. fig. 4) are seen within the clade of subtype C, in particular for the position of the *Bradyrhizobium*, *Inquilinus,* and *Zymomonas* proteins. The relatively low SH-like local support values refer to the selection of 39 taxa used to build the tree shown; much stronger values were found for the same nodes by using wider selections of taxa (cf. supplementary fig. S1, Supplementary Material online). (*B*) Structural differences in *cydA* proteins correlate with the new classification of *bd* oxidases. Selected sequences of *cydA* proteins from all (sub)types of *bd* oxidases were first aligned with the program COBALT after a wide DELTABLAST search (cf. fig. 1); the alignment was then manually refined in the regions of poor sequence identity, removing gaps in the predicted transmembrane segments (cf. Degli Esposti et al. 1993). The alignment blocks containing the invariant hem ligands of *bd* oxidases (Mogi 2009; Borisov et al. 2011) were then extracted and compacted together, separated by narrow gray gaps and a yellow gap corresponding to the Q loop extension. Residue changes that are specific for each subtype of *bd* oxidases are highlighted as gray for CIO subtype A; pale blue for CIO subtype B; and yellow for CIO subtype C.

**Fig. 7.—:**
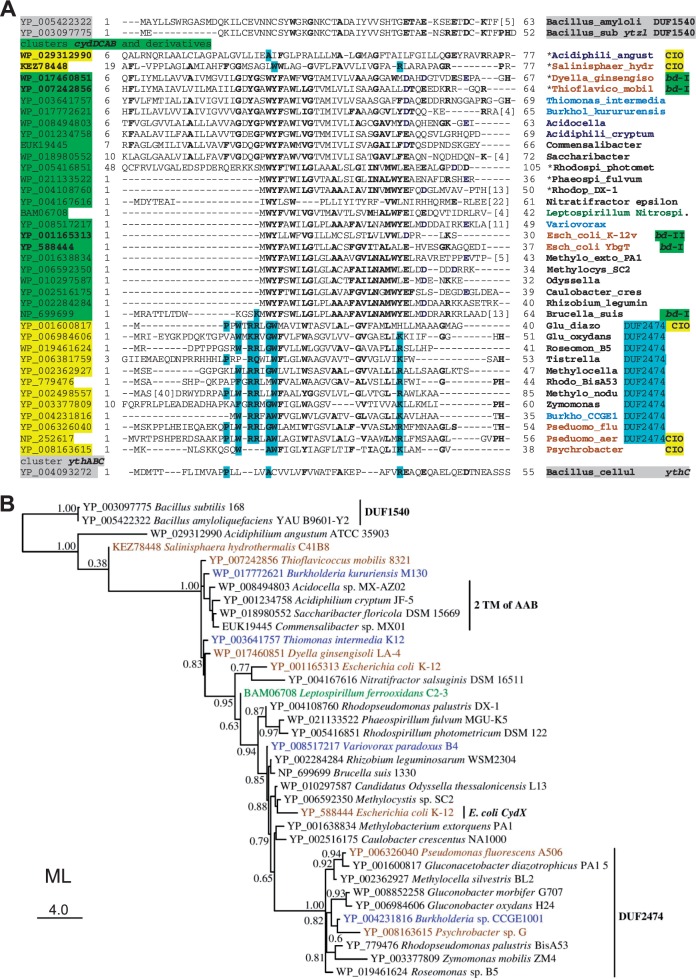
Alignment and phylogenetic tree of representative *cydX* and *cydX*-like subunits of *bd* oxidases. (*A*) Representative *cydX* sequences from the *bd* oxidases of *bd*-I type were first aligned to each other and subsequently to other hydrophobic proteins occupying the same appended position in the gene clusters of *bd* oxidases of CIO type (often possessing the DUF2474 domain, cf. fig. 2). The implementation of the overall alignment was guided by inspection of individual hydropathy profiles and the minimization of gaps in the predicted transmembrane segment, as described earlier for *COX*4 proteins (Degli Esposti et al. 2014). The top of the alignment contains representative sequences for DUF1540 proteins positioned as *cydX* in the *ythABC* clusters of ancestral *bd* oxidases from Firmicutes. Conserved residues are in bold characters and those typical of DUF2474 proteins are highlighted in pale blue. The symbol * indicates organisms with the *cydABDC* operon. Bacillus_amyloli, *Bacillus amydoliticus*; Bacillus_sub, *Bacillus subtilis*; Bacillus_cellul, *Bacillus cellulosilyticus*; Acidiphili_angust, *Acidiphilum angustum*; Salinisphaer_hydr, *Salinisphaera hydrothermalis*; Dyella_ginsengiso, *Dyella ginsengisoli*; Thioflavico_mobil, *Thioflavicoccus mobilis*; Burkhol_kurururensis, *Burkholderia kurururensis*; Acidocella, *Acidocella* sp. MX-AZ02; Acidiphili_cryptum*, Acidiphilium cryptum*; Commensalibacter, *Commensalibacter* sp. MX01; Saccharibacter, *Saccharibacter floricola*; Rhodospi_photomet, *Rhodospirillum photometricum*; Phaeospi_fulvum, *Phaeospirillum fulvum*; Rhodop_DX-1, *Rhodopseudomonas palustr*is sp. DX-1; Nitratifractor epsilon, *Nitratifractor salsuginis* DSM 16511—ε-proteobacteria; Leptospirillum Nitros., *Leptospirillum ferrooxidans* C2-3—Nitrospirales; Variovorax, *Variovorax paradoxus* B4; Esch_coli, *Escherchia coli*; Methylo_exto_PA1, *Methylobacterium extorquens* PA1; Methylocys_SC2, *Methylocystis* sp. SC2; Odyssella, *Ca. Odyssella thessalonicensis*; Caulobacter_cres, *Caulobacter crescentus* NA1000; Rhizobium_legumin, *Rhizobium leguminosarum* bv. *trifolii* WSM2304; Glu_diazo, *Gluconacetobacter diazotrophicus* PA1 5; Glu_oxydans, *Gluconobacter oxydans* H24; Roseomon_B5, *Roseomonas* sp. B5; Tistrella, *Tistrella mobilis* KA081020-065; Methylocella, *Methylocella silvestris* BL2; Rhodo_BisA53, *Rhodopseudomonas palustr*is sp. BisA53; Methylo_nodu, Methylobacterium nodulans; Zymomonas, *Zymomonas mobilis* subsp. *mobilis* ZM4 = ATCC 31821; Burkho_CCGE1, *Burkholderia* sp. CCGE1001; Pseduomo_flu, *Pseudomonas fluorescens* A506; Pseduomo_aer, *Pseudomonas aeruginosa* PAO1; Psychrobacter, *Psychrobacter* sp. G. (*B*) The ML tree was constructed on the basis of the manually curated alignment in (*A*) using the LG model (Le and Gascuel 2008). The unit of amino acid changes per branch is indicated on the bottom left, whereas SH-like local support values are shown adjacent to nodes.

Given the unclear picture of *bd* oxidase phylogeny (Brochier-Armanet et al. 2009; Borisov et al. 2011), we have followed complementary approaches to obtain integrated information that could support or adjust the results obtained with phylogenetic trees. Integrated information is particularly necessary to solve a difficult phylogenetic issue (Philippe and Roure 2011), such as the molecular evolution of *bd* oxidases and its entwined relationship with the phylogeny of proteobacteria. The first approach has been the systematic analysis of phylogenetic trees obtained with DELTABLAST (Boratyn et al. 2012) for subunit I of the oxidase enzyme, which is coded by the *cydA* gene (Winstedt et al. 1998). This protein binds cytochrome *b*-595 and cytochrome *d* (together forming the oxygen-reacting binuclear centre; Mogi 2009; Borisov et al. 2011), as well as cytochrome *b*-558 which directly reacts with (ubi)quinol (Borisov et al. 2011). Trees obtained with proteobacterial protein queries did not provide a coherent phylogenetic picture until valuable outgroup taxa could be found by extending DELTABLAST searches to the Firmicutes (Winstedt and von Wachenfeldt 2000) and Nitrospirales (Lücker et al. 2010), as shown in figures 1, 3, 4, and 6, as well as supplementary figure S1, Supplementary Material online. A parallel phylogenomic analysis (Segata et al. 2013) produced reference trees for α- and γ-proteobacteria (fig. 5 and supplementary fig. S2, Supplementary Material online) to verify the congruency of specific nodes in the trees obtained with the catalytic subunits of *bd* oxidases. The second approach was to match the results obtained from phylogenetic trees with the genomic structure of the *bd* oxidases (fig. 2), as previously used for classifying the operons of *aa_3_*-type oxidases (Degli Esposti et al. 2014). Systematic analysis of over 200 gene clusters generated a valuable set of characters, in the form of recurrent gene sequences or structure-functional similarities of the coded proteins, for the genomic regions surrounding the diad *cydA–cydB*, which constitutes the catalytic core of the enzyme (figs. 2, 3*B*, and 4*B*). *CydB* codes for subunit II of *bd* oxidases, shorter than subunit I and also essential for catalysis, even if it does not directly carry hem ligands (Borisov et al. 2011). A subsequent approach was to identify protein signatures for the newly classified subtypes of *bd* oxidases in refined alignments of representative sequences of the *cydA* subunit (fig. 6*B*). Finally, we evaluated the sequence similarity and hydrophobicity profile of the small subunit of *bd* oxidases, generally called *cydX* (Hoeser et al. 2014). This short hydrophobic subunit has been recently identified in *bd*-I type oxidases (Sun et al. 2012; VanOrsdel et al. 2013; Hoeser et al. 2014), but we have found similar proteins also within the gene clusters of CIO type oxidases (fig. 7).

### Approach 1—Resolving the Phylogenetic Trees for *bd* Oxidases

The taxonomic distribution of *bd* oxidases is very broad, encompassing at least two Archean *phyla* and diverse groups of Eubacteria, from Gram-positive Firmicutes and Actinomycetes to the whole *phylum* of proteobacteria (Brochier-Armanet et al. 2009). In previous studies, groups of Gram-positive taxa have been found to cluster together with some proteobacterial lineages, thereby producing phylogenetic trees that are clearly incongruent with accepted evolutionary patterns (Hao and Golding 2006; Brochier-Armanet et al. 2009). To complicate the picture further, the genomes of several α-, β-, and γ-proteobacteria, as well as of some strains of *Bacillus* (Winstedt and von Wachenfeldt 2000), contain two or more gene clusters of *bd* oxidases with different molecular features in the catalytic subunits (Borisov et al. 2011; Degli Esposti et al. 2014) that have not been considered in previous phylogenetic studies (Hao and Golding 2006; Brochier-Armanet et al. 2009). Therefore, the same bacterial taxon could easily appear in different clades of phylogenetic trees for either *cydA* or *cydB* due to the independent clustering of its multiple forms of *bd* oxidases.

In our systematic approach of analyzing *bd* oxidases, we first undertook a survey of all their *cydA* proteins that are currently present in the NR databank, which amount to approximately 9,200 genuine entries for proteobacteria alone (i.e., without isolated fragments that are common in enterobacterial genomes). Among these entries we then identified the major types of *bd* oxidases on the basis of their sequences; initially, we followed the empirical rule of categorizing them as *bd*-I type whenever the length of their *cydA* subunit exceeded 500 resides (cf. Degli Esposti et al. 2014). Subsequently we verified whether the *cydA* proteins contained a long extension in the so-called Q loop, for which we had prepared a manually curated alignment (not shown). We additionally evaluated the length of the associated *cydB* for the same type of enzyme and performed specific BLAST analysis of both subunits when their length or features did not match the common pattern.

Once the categorization of all proteobacterial sequences was completed (see supplementary table S1, Supplementary Material online), the organisms that possess only one *bd*-I type oxidase such as *Brucella* (Sun et al. 2012) were separately considered from those possessing two similar forms of *bd*-I type oxidase such as *E. coli* (Borisov et al. 2011) or *Methylocystis* (Degli Esposti et al. 2014) and those possessing both the *bd*-I type and the CIO type of oxidase, such as several Rhodospirillales (Degli Esposti et al. 2014). Conversely, taxa that possess only one CIO type oxidase such as *Rickettsia* were categorized differently from those presenting two or more forms of CIO type, for example, several species of the *Methylobacterium* genus (supplementary table S1, Supplementary Material online). We thus built a compilation of bacterial taxa according to the type and number of *bd* oxidases they possess. Such compilation was subsequently used to analyze the phylogenetic trees obtained with DELTABLAST searches over selected databases, enabling the distinction of bacteria that characteristically have a single type of *bd* oxidase from those having multiple types and/or forms of the enzyme.

Although no clear pattern could be discerned in the distribution of the two types of *bd* oxidases among the classes of α-, β-, and γ-proteobacteria, we realized that ε-proteobacteria possess only the *bd*-I type. Conversely, the majority of δ-proteobacteria has short *cydA* and *cydB* proteins that are evidently related to the catalytic subunits of ancestral *bd* oxidases of *Bacillus subtilis* (Winstedt et al. 1998). A dozen δ-proteobacteria including *Desulfovibrio magneticus* (Nakazawa et al. 2009) and *Desulfococcus multivorans* (Brown et al. 2013) have another *bd* oxidase, which clusters with the CIO type of α-, β-, and γ-proteobacteria, as illustrated in figure 1. Furthermore, a phylogenetically different group of δ-proteobacteria such as *Desulfobulbus propionicus* has *cydA* sequences that are longer than those of the rest of the class, as they possess Q loop extensions similar to those of *bd*-I type oxidases. In addition, the genomes of over 50 δ-proteobacteria contain chimaeric forms of *cydA* that are fused with multiple *c*-type cytochromes and are not associated with a *cydB* gene; consequently, they do not correspond to proper *bd* oxidases. These unusual proteins were then discarded from our analysis.

The diversity of molecular forms of *bd* oxidases in δ-proteobacteria has not been recognized earlier and significantly contributes to complicate the molecular evolution of this enzyme, together with the abundance of gene sequences from Enterobacteria. Indeed, approximately one-half of all the proteobacterial *bd* sequences that are present in the NR database belong to this group of γ-proteobacteria (Gao et al. 2009; Williams et al. 2010) with similar representation of the *bd*-I and CIO type (supplementary table S1, Supplementary Material online; and Rosas-Pérez T, Degli Esposti M, unpublished data). After analyzing the taxonomic distribution of these types and determining the strong similarity of enterobacterial *cydA* and *cydB* proteins, we decided to use only a few representative species of Enterobacteria in subsequent studies, in order to reduce sequence redundancy and widen the phylogenetic breadth of our analysis.

We next considered the most appropriate taxa to root the phylogenetic trees of our systematic analysis of the core catalytic proteins of *bd* oxidases. A survey of the growing literature on α-proteobacterial phylogeny revealed a recurrent problem that we wished to avoid, for it can affect the overall topology of phylogenetic trees: The selection of β- and/or γ-proteobacteria (including *E. coli*) as outgroup to root the trees (Viklund et al. 2012; Luo et al. 2013; Ferla et al. 2013; Amrine et al. 2014). This choice may constitute a problem because β- and γ-proteobacteria separated from the ancestral α-proteobacterial lineage (Woese 1987) and consequently cannot produce a solid root of α-proteobacterial taxa that evolved in parallel, if not before them. We found that Firmicutes represent a valuable taxonomic group for rooting phylogenetic trees of *bd* oxidases not only for their ancestral forms of the enzyme but also considering the abundant information that is available on the genetic and biochemical properties of their oxidases (Winstedt et al. 1998; Azarkina et al. 1999; Sakamoto et al. 1999; Winstedt and von Wachenfeldt 2000). When wide (i.e., extended to 1,000 or more taxa) DELTABLAST searches include Firmicutes and the query is chosen among the *cydA* sequences of *B. subtilis, Nitrospira,* δ-proteobacteria, ε-proteobacteria, or *Acidocella*, the structure of phylogenetic trees is robust and reproducible, as shown in figure 1 and supplementary figure S1, Supplementary Material online. These trees clearly define a major split between the CIO type and the *bd*-I type of proteobacterial *bd* oxidases, the latter type descending from a subset of *Bacillus* proteins that belong to the cytochrome *bd* oxidase characterized by Winstedt et al. (1998)—with *cydA* accession: NP_391755. Sequences from δ-proteobacteria predominantly cluster between the *Bacillus* sequences and the major split between the two types of *bd* oxidases; instead, those of ε-proteobacteria and of the *Leptospirillum* group of Nitrospirales aggregate with the *bd-*I type whereas that of *Nitrospira* associates with the CIO type (figs. 1 and 3). This tree topology has been confirmed with diverse programs of phylogenetic analysis (figs. 1 and 3*A* and supplementary fig. S1, Supplementary Material online).

The results shown in figure 1 produce a comprehensive resolution of the molecular evolution of the *bd* oxidases present in proteobacteria. Previously published phylogenetic trees (Hao and Golding 2006; Brochier-Armanet et al. 2009) had generated a partial picture of this evolution (cf. Borisov et al. 2011) owing, in particular, to the lack of recognition of the diverse types, and multiple forms per single taxon, of the catalytic proteins of the oxidase enzyme.

### Approach 2—Analysis of the Gene Clusters of *bd* Oxidases

We next integrated the above phylogenetic data with a detailed analysis of the gene clusters of *bd* oxidases, the common patterns of which are graphically illustrated in figure 2. The ancestral, prototypic gene cluster corresponds to the *cydABDC* operon of *Bacillus subtilis* (Winstedt et al. 1998). In its original sequence, it is preserved in the gene clusters of the *bd-I* type oxidases of some α-proteobacteria belonging to the family of Bradyrhizobiaceae and Rhodospirillaceae plus *Pleomorphomonas oryzae* (Xie and Yokota 2005), as shown in figure 3*B*. The latter bacterium is tentatively classified within the family of Methylocystaceae, the other members of which show a split of the original operon into two separate gene clusters as in *E. coli*, that is, c*ydABX* (VanOrsdel et al. 2013) and *cydDC* (Borisov et al. 2011). However, many Rhizobiales, Rhodospirillales, and Caulobacterales possess a modified version of the ancestral operon in which the genes for the cysteine/glutathione transporters, *cydD* and *cydC*, are transposed in prepended position with respect to the catalytic subunits (figs. 2 and 3*B*), thus forming the same *cydDCAB* operon as that of *Brucella* (Sun et al. 2012). Outside α-proteobacteria, this modified cluster appears to be present in the Firmicute *Listeria innocua* (protein accession: WP_003772850), some β-proteobacteria such as *Variovorax paradoxus B4* (gene accession: VAPA_1c34620) and two γ-proteobacteria*, Salinisphaera hydrothermalis* (fig. 3*B*) and *Psychrobacter* sp. PRwf-1.

The original sequence of the *cydABDC* operon is present also in the *bd*-I type of a few other proteobacterial taxa (fig. 3*B*): A single δ-proteobacterial organism, *Desulfurivibrio alkaliphilus*, the γ-proteobacteria *Dyella ginsengisoli, Frateuria aurantia,* and *Thioflavicoccus mobilis* (fig. 3*B*), as well as the β-proteobacterial endosymbiont, *Kinetoplastibacterium blastocrithidii* (Alves et al. 2013). Nevetheless, α-proteobacteria retain the largest number of original or partially modified operons for *bd*-I type oxidases, a surprising finding considering that these enzymes have been well studied in *E. coli* (Mogi 2009; Borisov et al. 2011), which has not retained the ancestral operon (fig. 3*B*). Of note, the protein sequence of the catalytic subunits of *bd*-I type oxidases has remained largely unaltered in proteobacteria, thereby producing a compact topology in phylogenetic trees (fig. 3*A*).

Intriguingly, the gene cluster for the CIO type oxidase of *Acidocella* and *Acidiphilium angustum* also corresponds to the *cydABDC* operon (figs. 2 and 4*B*), a situation that applies also to the following γ-proteobacteria: *S. hydrothermalis*, *Halothiobacillus neapolitanus,* and a few organisms of the enterobacterial genera *Tatumella* and *Pantoea* (fig. 1). Modified forms of the original gene clusters are additionally present in *Defluviimonas* sp. 20V17, a marine α-proteobacterium of the *Roseobacter* group (Jiang et al. 2014) which usually does not possess *bd* oxidases (Degli Esposti et al. 2014), and the γ-proteobacterium *Thiobacillus prosperus*. Other proteobacteria do not show such gene clusters for CIO type *bd* oxidases, thereby implying that β- or δ-proteobacteria, as well as many α- and γ-proteobacteria, have either completely lost the ancestral *cydABDC* operon or acquired a different gene cluster from their progenitors. The *ythABC* operon coding for the second form of *bd* oxidase in *Bacillus* (Sakamoto et al. 1999; Winstedt and von Wachenfeldt 2000) is the best candidate for such an alternative source of CIO type oxidases. In resolved phylogenetic trees, *ythA* is consistently placed upstream of *cydA* (figs. 1 and 3*A*), thereby supporting the possibility that proteobacteria separately inherited this protein and its associated operon from relatives of current *Bacillus* species, with subsequent differential distribution along evolution.

### New Classification of CIO Type *bd* Oxidases

The considerations just mentioned regarding the potentially different origin of CIO type *bd* oxidases suggest that the evolutionary history of these enzymes in proteobacteria could be retraced from detailed phylogenetic trees of their catalytic subunits. To verify this possibility, we enlarged the DELTABLAST searches of *cydA* proteins to 5,000 taxa, including only a few representative of δ-proteobacteria and excluding Enterobacteria for the reasons discussed earlier (supplementary fig. S1, Supplementary Material online). The overall tree topology of the results obtained (supplementary fig. S1, Supplementary Material online) essentially matched that of figure 1, showing an early separation of ancestral CIO sequences inserted in the original *cydABDC* operon, for example, *Acidocella* (accession: WP_008494833). This group has been named subtype A, with A indicating their likely ancestral nature. All other CIO sequences segregate in two sister branches which we have named subtype B (for the predominance of β-proteobacteria) and subtype C (for the inclusion of bona fide CIO oxidases such as those of *Pseudomonas*) that may derive from the *ythABC* operon of *Bacillus* (fig. 4 and supplementary fig. S1, Supplementary Material online). We thus introduce a new classification of CIO *bd* oxidases into three separate subtypes, A, B, and C, each of which contains *cydA* sequences from different lineages, as described below (supplementary fig. S1, Supplementary Material online).

Subtype A contains a restricted group of α-proteobacteria related to *Acidocella* together with a set of γ-proteobacteria that in general belong to deep branches of their class, as in the case of *S. hydrothermalis* (supplementary fig. S2, Supplementary Material online, see also Zhang et al. 2012). Subtype B contains several β-proteobacteria such as *Deefgea rivuli* (Stackebrandt et al. 2007) and a characteristic group of α-proteobacteria including *Micavibrio*, *Midichloria*, *Mesorhizobium,* and *Rhodospirillum centenum* (with its 448 residues long *cydA*, accession: YP_00226549; fig. 4 and supplementary fig. S1, Supplementary Material online). The single *bd* oxidase of other Rhodospirillales such as *Eliorea* also falls under subtype B, which additionally contains an assortment of Rhodobacterales and Rhizobiales that possess a *bd*-I type oxidase, too. Finally, subtype B includes γ-proteobacteria (fig. 4) belonging to the Oceanospirillales, for instance, *Oceanospirillum beijerinckii* (fig. 4).

By far, subtype C contains the largest set of *cydA* proteins and taxa among CIO subtypes (fig. 4 and supplementary fig. S1, Supplementary Material online). It includes β-proteobacteria such as those shown near to subtype A clade in supplementary figure S1, Supplementary Material online, and a small group of δ-proteobacteria, as previously mentioned (fig. 1). However, the great majority of species harboring subtype C oxidases belongs to γ- and α-proteobacteria (fig. 4 and supplementary fig. S1, Supplementary Material online), several of which have two *cydA* proteins that fall under subtype C (supplementary table S1, Supplementary Material online), most likely derived from recent processes of gene duplication. The deep separation between the groups of subtypes A and B (fig. 4) suggests instead an earlier process of duplication of the ancestral operon related to *ythABC* of *Bacillus*. This concept is supported by the finding that some taxa possess CIO *bd* oxidases of both subtypes A and B: The Rhodospirillales *R. centenum* and *Roseomonas cervicalis*, the Bradyrhizobiaceae *Salinarimonas rosea* and *Bradyrhizobium diazoefficiens*, and the β-proteobacteria *Cupriavidus metallidurans*, *Ralstonia* sp. AU12/08, and *Bordetella petrii*. The presence of both subtypes B and C in the same bacterial genome is thus relatively rare compared with the large number of subtype C proteins, suggesting that such a situation may be the relic of a primordial stage in the evolution of the *bd* oxidases of CIO type. Therefore, the classification of these oxidases we introduce here (fig. 4 and supplementary fig. S1, Supplementary Material online) provides novel information for interpreting the molecular evolution of the enzyme. Does it provide also clues for the evolution of proteobacteria that possess *bd* oxidases? In the case of Rickettsiales, the answer may well be positive, as discussed below.

### Rickettsiales Have Unusual CIO *bd* Oxidases Possibly Related to Some γ-Proteobacteria

Approximately one-half of the available genomes for the *Rickettsia* genus and a fraction of those for *Wolbachia* present gene clusters encoding *bd* oxidases of CIO type (McLeod et al. 2004; Degli Esposti et al. 2014). The *cydA* proteins from these species cluster in a separate subtype C branch which includes also the sequences of two γ-proteobacterial genera, *Psychrobacter* and *Cycloclasticus* (fig. 4 and supplementary fig. S1, Supplementary Material online). *Cycloclasticus* is a genus of marine bacteria capable of oxidizing hydrocarbons (Lai et al. 2012), whereas *Psychrobacter* belongs to the family of Moraxellaceae, which contains pathogenic organisms such as *Moraxella catarrhalis* and *Acinetobacter baumannii* (Pettersson et al. 1998). Intriguingly, the *bd* oxidase gene cluster of *Psychrobacter* sp. JCM 18902 and *Psychrobacter* sp. G (Kudo et al. 2014) shows the outer membrane protein *Imp*, which is required for cell envelope biogenesis (Braun and Silhavy 2002), exactly in the same appended position in which the gene cluster of *Rickettsia conorii* and related species have another protein involved in cell envelope biogenesis*,* the beta-1,4 glucosyltransferase *wcaA* (Stevenson et al. 1996). Moreover, the gene clusters of both *Rickettsia* and *Psychrobacter* contain proteins involved in defense mechanisms, which are rarely found in clusters of other bacteria.

These similarities between the *bd* oxidase of *Rickettsia* and *Psychrobacter* contrast with the evidence that *Midichloria*, an early-branching member of the Rickettsiales order (Sassera et al. 2011), possesses a completely different gene cluster and a *cydA* that clearly segregates with subtype B rather than subtype C (fig. 4). Conversely, an endosymbiont of *Acanthamoeba* that has been reported to be related to *Midichloria* (Wang and Wu 2014) possesses a gene cluster and *cydA* protein that are close to those of *Rickettsia* but clearly separate from those of *Midichloria* (fig. 4 and data not shown). It is therefore intriguing that a single ancestral member of the Rickettsiales, *Midichloria*, has a CIO *bd* oxidase that is different from all other members of the same order. The simplest explanation would be to invoke a case of LGT just for *Midichloria*. However, we favor the alternative possibility that *Midichloria* has uniquely retained one of the duplicated versions of the ancestral CIO *bd* oxidase (subtype B) that was present in organisms in which such duplication had occurred early during evolution, whereas the remaining Rickettsiales have inherited the other duplicate of CIO oxidase that falls into subtype C. This possibility would imply that the free-living ancestors of all Rickettsiales were related to nonnodulating members of the Bradyrhizobiaceae family such as *Salinarimonas* (Liu et al. 2010) or Rhodospirillales such as the pathogenic *Ro. cervicalis* (Rihs et al. 1993) that maintain *bd* oxidases of both subtypes B and C, as mentioned earlier.

### Clear Cases of LGT for *bd* Oxidases

Inspection of enlarged trees for CIO of subtype C (cf. supplementary fig. S1, Supplementary Material online) has revealed one evident case of LGT. *Bartonella tamiae* (Kosoy et al. 2008) is the only member of the Rhizobiale family of Bartonellaceae possessing a *bd* oxidase (Degli Esposti et al. 2014), but the sequences of its *cydA* (fig. 4*A*) and *cydB* (fig. 6 below) proteins are very close to those of the phylogenetically unrelated *Asticcacaulis benevestitus*, which belongs to the Caulobacterales (Vasilyeva et al. 2006). The phylogenetic distance of these organisms emerges clearly from trees constructed with phylogenomic approaches (fig. 5*A*). Another clear event of LGT regards the entire *cydABDC* operon for the *bd* oxidases of subtype A that is present in some enterobacterial organisms of the genera *Pantoea* and *Tatumella* (fig. 1), which cluster with *E. coli* in the most recent branch of the phylogenetic tree of γ-proteobacteria (supplementary fig. S2, Supplementary Material online). Other γ-proteobacteria having the *cydABDC* operon lie instead in basal branches of the same tree (supplementary fig. S2, Supplementary Material online). The ecological and microbiological features of these taxa do not provide clues for rationalizing the suspected LGT event. However, the fact that the core *cydABDC* operon for *bd* oxidases of CIO type is present also in phylogenetically diverse taxa of α-proteobacteria, namely the Rhodospirillale *Acidocella* and the Rhodobacterale *Defluviimonas* (fig. 1 and supplementary fig. S1, Supplementary Material online), suggests that this operon may have features that facilitate its lateral transmission among proteobacteria. Ancestors of the extant *Acidocella* would have been the donor organisms for such events of LGT, as indicated by data presented later.

### Phylogenetic Congruency and the Molecular Evolution of *bd* Oxidases

The above considerations regarding cases of LGT suggested to evaluate in depth the congruency of the current taxonomic distribution of CIO type *bd* oxidases (fig. 4 cf. supplementary fig. S1, Supplementary Material online) with respect to the evolutionary pattern of α- and γ-proteobacteria outlined by studies of phylogenomics (Williams et al. 2007, 2010; Brochier-Armanet et al. 2009; Gao et al. 2009; Sassera et al. 2011; Segata et al. 2013). To this end, we undertook a detailed phylogenomic analysis of the 40 taxa used in the representative phylogenetic trees of *bd*-I type (fig. 3) and CIO type (fig. 4) oxidases and constructed a tree of reference from the results obtained (fig. 5*A*). In such a tree, α-proteobacteria segregate in a separate clade from that containing β- and γ-proteobacteria, as expected, whereas Rickettsiales occupy a strongly supported basal position, which is consistent with previously published trees (Williams et al. 2007; Ferla et al. 2013). However, *Nitrospira defluvii*, the representative member of the Nitrospirales group (Lücker et al. 2010), clusters with the branch of α-proteobacteria and not at the basis of all proteobacteria, as expected and indeed regularly found in phylogenetic trees of CIO type *bd* oxidases (fig. 4 and supplementary fig. S1, Supplementary Material online). After removing the Rickettsiales from the phylogenomic analysis, *Nitrospira* segregated with the branch containing β- and γ-proteobacteria, whereas *Zymomonas* appeared to be the most basal taxon of α-proteobacteria. Clearly, the presence of Rickettsiales strongly influences the topology of phylogenetic trees constructed with phylogenomic approaches, altering the pattern of ancestral evolution of proteobacteria versus Nitrospirales. This situation reflects insuppressible distortions derived from long-branch attraction phenomena in the genomes of obligate endocellular parasites such as Rickettsiales (Williams et al. 2010; Philippe and Roure 2011).

To minimize these distortions and verify the possible evolutionary pattern of α-proteobacteria with respect to the emerging evolution of *bd* oxidases (cf. figs 1 and 4), we then analyzed the Rieske iron-sulfur subunit of ubiquinol-cytochrome *c* reductase, hereafter abbreviated as ISP. This bioenergetic protein contains strong phylogenetic signals (Degli Esposti et al. 2014) and performs the same basic function as *bd* oxidases, namely oxidation of ubiquinol. Another advantage of using ISP in the phylogenetic comparison with *bd* oxidases is that it has two forms in α-proteobacteria, the shorter or ISP1 being equivalent to mitochondrial ISP whereas the longer or ISP2 is closely related to its homologues in β- and γ-proteobacteria (Degli Esposti et al. 2014). It is thus possible to verify the phylogenetic vicinity of β- and γ- to α-proteobacteria on the basis of the sequence similarity of their ISP. The results of our analysis indicate that *Acidothiobacillus* and *Halothiobacillus* (fig. 5*B*), as well as *Legionella* and *Frateuria* (not shown), are the closest γ-proteobacterial genera to α-proteobacterial species with ISP2, whereas the β-proteobacteria previously considered here (figs. 3 and 4) lie in distant branches (fig. 5*B*). Although the ISP of these γ-proteobacterial genera clusters in basal branches of their class (supplementary fig. S2, Supplementary Material online), *Acidocella* ISP is consistently placed in the most distant clade of all proteobacteria (fig. 5*B* and data not shown). (Of note, *Acidiphilium* does not possess ISP [San Martin-Uriz et al. 2011] and thus its phylogenetic position cannot be visualized in these trees).

Importantly, phylogenetic trees constructed with ISP sequences show Rickettsiales in a later-branching clade of the ISP1 form, consistent with recently published data (Degli Esposti et al. 2014) and the later-branching position of Rickettisales in phylogenetic trees of the *cydA* proteins (fig. 4 and supplementary fig. S1, Supplementary Material online). The same topology is obtained in phylogenetic trees of *cydB* proteins (fig. 6), which closely match those of *cydA* proteins (fig. 4). The latter result rectifies a previous study that suggested differential rates of evolution for the two catalytic subunits of *bd* oxidases (Hao and Golding 2006). *CydB* trees also confirm the basal position of *Parvibaculum* CIO oxidase among those of subtype C (figs. 4*A* and 6), a situation consistent with the proposed ancestral nature of this α-proteobacterium (Schleheck et al. 2011; Viklund et al. 2012), even if it is not confirmed in phylogenomic trees (fig. 5*A*).

### Protein Signatures of CIO Subtypes of *bd* Oxidases

The new classification of CIO type *bd* oxidases (fig. 4 and supplementary fig. S1, Supplementary Material online) can be correlated with specific protein signatures in the catalytic regions of the *cydA* subunit, as shown in figure 6*B*. The N-terminal part of the protein contains the binding pocket of oxygen-reacting cytochrome *b*-595, in which the haem is ligated to invariant His19 (*E. coli* numbering; Borisov et al. 2011). The residues surrounding this residue are highly conserved in the sequences of *bd*-I type oxidases but show distinctive variations in the sequences of CIO type oxidases (Mogi 2009), in particular those of subtype A (highlighted in gray in fig. 6*B*) and subtype B (highlighted in pale blue in fig. 6*B*). Additional variations of conserved residues appear to be present in the sequences of subtype C proteins (highlighted in yellow in fig. 6*B*), which retain six changes specific to subtype A together with five conserved residues of *bd*-I type sequences (fig. 6*B*). A similar pattern of amino acid substitution is found around the invariant Glu99 that is considered the prime candidate for ligating oxygen-reacting hem *d* in *bd* oxidases (Mogi 2009; Borisov et al. 2011).

The region surrounding His186, one of the hem ligands of quinol-reacting cytochrome *b*-558 in *bd* oxidases (Borisov et al. 2011), also changes between *bd*-I and CIO type sequences; of the nine conserved residues found in *bd*-I proteins, three are retained in subtypes A and C and only one in subtype B (fig. 6*B*, middle). Remarkable amino acid substitutions occur in the positions adjacent to the histidine ligand, with Val185 changing to Pro in subtype A and some proteins of subtype C, whereas Thr187 is substituted by Met in all CIO subtypes (fig. 6*B*). By analogy with cytochrome *b* of the *bc_1_* complex (Degli Esposti et al. 1993; Gao et al. 2003), these substitutions significantly alter the local volume packing of the protein and may induce resistance to quinone-antagonist inhibitors such as hydroxyquinoline-N-oxides (e.g., HQNO), which inhibits both *bd* oxidases and the *bc_1_* complex (Borisov et al. 2011 cf. Degli Esposti et al. 1993). The same applies to amino acid changes one helical turn away from the ligand histidine, such as Ala182 in *bd*-I oxidases becoming Tyr in subtype B CIO oxidases and Lys183 in *bd*-I oxidases becoming Gly in *Acidocella* subtype A CIO oxidase (fig. 6*B*, middle). Conversely, the region surrounding Met393, the other hem ligand in cytochrome *b*-558 (Borisov et al. 2011), shows hardly any variation across all (sub) types of *bd* oxidases (fig. 6*B*, right).

### Sequence Analysis of the Short Subunit of *bd* Oxidases, *cydX*

Besides the two catalytic subunits, gene clusters of several *bd* oxidases contain a third small subunit, *cydX* (Sun et al. 2012; VanOrsdel et al. 2013; Hoeser et al. 2014). This subunit contains a transmembrane segment that starts with highly conserved aromatic residues and a C-terminus predicted to be exposed at the periplasmic side of the membrane (Sun et al. 2012; Hoeser et al. 2014); its sequence in *E. coli* has been categorized under the PRK14749 superfamily restricted to Enterobacteria (Marcher-Bauer et al. 2011). It has been recently demonstrated that *cydX* copurifies with the *bd*-I oxidase of *E. coli* and is essential for its catalytic activity, as its deletion leads to the loss of the oxygen-reacting hems (VanOrsdel et al. 2013; Hoeser et al. 2014). Here, we extended previous analyses of *cydX* by aligning all the small hydrophobic proteins appended to *cydB* in the genes clusters of *bd* oxidases (cf. figs. 2–4) and found that they show local sequence similarity with two families of Domain of Unknown Function (DUF; Goodacre et al. 2013), which also have a single transmembrane segment: DUF1540 in Firmicutes (fig. 7*A*, top) and DUF2474 in proteobacteria (fig. 7*A*, bottom). In the latter case, the small hydrophobic protein is associated with CIO oxidases of subtype C, thereby producing a new character for this subtype of *bd* oxidases. In subtype B, only the gene cluster of *Midichloria* shows a short protein downstream of *cydB* (fig. 4*B*), which has features of signal peptides but no predicted transmembrane segment, similarly to a protein that lies in the same appended position of the gene cluster of *Acidothiobacillus caldus* CIO (accession: WP_014002942). Signal features include the Twin arginine translocase (Tat) motif that is conserved in the N-terminal part of DUF2474 proteins (fig. 7*A*).

In the genomic position just downstream of *cydB*, the CIO type oxidases of *Acidiphilum angustum* and *S. hydrothermalis* have a hydrophobic protein of 77 residues with two predicted transmembrane segments, the second of which shows clear sequence similarity with the *cydX*-like proteins of acetic acid bacteria (fig. 7*A*, top). These proteins are also predicted to have two transmembrane segments (cf. fig. 2) and align well to the shorter *cydX* sequence of *E. coli* or other proteobacteria (fig. 7*A*). Interestingly, *cydX* of *Brucella* (Sun et al. 2012) has an N-terminal extension that can be partially aligned to the first transmembrane segment of the proteins from acetic acid bacteria or DUF2474 proteins, which generally have a long N-terminal extension and a truncated C-terminus with respect to *cydX*. DUF2474 proteins show specific conserved residues at the beginning of the predicted transmembrane segment, including a conserved Tat motif suggestive of protein export (highlighted in pale blue in fig. 7*A*). Hence, the membrane topology of DUF2474 would be the same as that predicted for *E. coli cydX* (Sun et al. 2012; Hoeser et al. 2014).

In sum, our taxonomically broad alignment (fig. 7*A* and data not shown) indicates that nearly all the small hydrophobic subunits that share the same appended position in the gene clusters of *bd* oxidases possess the common element of a single transmembrane segment flanked by relatively conserved residues, predominantly aromatic toward the N-terminus and charged toward the C-terminus, which is exposed at the periplasmic side of the plasma membrane. This simple structure is maintained from the y*thC* proteins of *Bacillus* to the DUF2474 proteins of *Pseudomonas* (fig. 7*A*) and is reminiscent of that previously found for *COX*4 in the gene clusters of *aa_3_*-type oxidases (Degli Esposti et al. 2014). The obvious question regarding both *cydX* and *COX*4 is why they are not present in all the gene clusters of closely related taxa. For instance, the *bd*-I type of *Tistrella mobilis* does not show this protein, contrary to the other oxidases of the family Rhodospirillaceae. One explanation is that such small proteins can be easily missed in standard genomic analysis for identifying and annotating open reading frames (Hoeser et al. 2014). Alternatively, the gene for these proteins might have migrated to other parts of the genomes, as in the case of *cydD* of the ancestral *cydABDC* operon that was mentioned earlier. Moreover, most members of the DUF1540 family are found away from the gene clusters of Firmicute *bd* oxidases. Therefore, the apparent absence of *cydX* or related proteins appended to the catalytic subunits of *bd* oxidases is not a definite proof for their absence from the enzyme complex, in which its function seems now established (Hoeser et al. 2014).

An interesting observation arises from the alignment in figure 7*A*. The *cydX*-like protein of the γ-proteobacterium *S. hydrothermalis* aligns to either the similarly long proteins of *bd*-I type oxidases from acetic acid bacteria or to DUF2474 proteins (fig. 7*A* and data not shown). This may indicate that the protein is related to both the long version of *cydX* with an additional transmembrane segment and to the DUF2474 proteins that have lost most of the hydrophobic part of this segment in their N-terminal extension, but could still function as a signal peptide for membrane translocation. To test this further, we have produced ML phylogenetic trees from the manually curated alignment of figure 7*A*, which matched those for the catalytic subunits of the *bd* oxidases (cf. fig. 1) in that they show the protein of *S. hydrothermalis* CIO in the second most basal branch for proteobacteria, just after that of *A. angustum* (fig. 7*B*). Consequently, these proteins can be considered as the likely progenitors of all *cydX* and related proteins such as DUF2474 that are found in the gene clusters of *bd* oxidases—except for the highly divergent DUF1540 from *Bacilli* (fig. 7). We then propose to rename all the proteins aligning to *cydX* as *cydX*-like.

## Conclusions and Perspectives

This work presents a comprehensive analysis of the molecular complexity of proteobacterial cytochrome *bd* oxidases, a broadly distributed family of enzymes involved in energy production and other functions that are essential to bacteria (Borisov et al. 2011; Giuffrè et al. 2014). Such functions include resistance to heavy metals, detoxification of oxygen or nitrosyl radicals and responses to various stress conditions, including engulfment by phagocytic cells. So far, functional and microbiological studies have been predominantly conducted in enteric and pathogenic proteobacterial taxa such as *Escherichia coli* and *Brucella* (Sun et al. 2012; Giuffrè et al. 2014), which have *bd*-I type oxidases. Outside a few organisms such as *Pseudomonas*, *Azotobacter,* and *Zymomomas,* very little is known of CIO type *bd* oxidases. Hence, it would not be surprising to find, eventually, that the newly classified *bd* oxidases of one subtype fulfill a specific physiological function. However, CIO *bd* oxidases of subtypes A and B have yet to be characterized biochemically, thus creating new perspectives for future studies on these enzymes. For example, the structure–function correlations we have uncovered in the quinol reacting regions of subunit I (fig. 6*B*) may guide the search of pharmacologically relevant inhibitors of the enzyme.

The variations in the haem-binding regions of *bd* oxidases (fig. 6*B*) suggest that our classification (figs. 1 and 4 and supplementary fig. S1, Supplementary Material online) bears significance to functional properties that are related to the evolution of the enzymes, in particular from strictly anaerobic to aerobic environments. Such an evolution has included adaptation to the ubiquinol substrate that is predominant in *Acidocella* and other α-proteobacteria (Jones et al. 2013). Instead, in Firmicutes and anaerobic proteobacteria the donor substrate is the low-potential quinol, menaquinol (Borisov et al. 2011). This functional consideration may explain the association of three genes for ubiquinone biosynthesis in the *bd* gene cluster of *D. magneticus* (accession: DMR_06960) and related δ-proteobacteria which segregate with CIO type oxidases (fig. 1). Similar biosynthetic genes have been found only in one other gene cluster for *bd* oxidases that is contained in the genome of *Psychrobacter*. Our systematic efforts of classification have thus produced a novel piece of phylogenetic information for the molecular evolution of *bd* oxidases. The *bd*-I type, which is specialized in oxidizing quinols under occasional conditions of extreme anaerobiosis (Borisov et al. 2011), originated from a duplication event in primordial proteobacteria that has left a clear trace in the genome of extant *Acidocella* and in the sporadic presence of the ancestral operon of Firmicutes in various proteobacterial taxa.

In concluding the analysis of the core subunits of *bd* oxidases, it is interesting to note how frequently the proteins of *Acidocella* lie in basal branches of the phylogenetic trees encompassing the whole *phylum* of proteobacteria (figs. 4, 5*B*, 8*B*, and 9*B*). These organisms are classified under the α-proteobacterial family of Acetobacteraceae, even if they possess genomic and functional features that are not typical of representative acetic acid bacteria such as *Acetobacter* (Jones et al. 2013; Servín-Garcidueñas et al. 2013)*. Acidocella* strains are closely related to one species of the genus *Acidiphilium*, *A. angustum*, while showing genomic features distinguishing them clearly from other *Acidiphilum* organisms. For example, *Acidocella* and *A. angustum* have two separate *cydABDC* gene clusters for *bd* oxidases, one identical to the ancestral operon of *Bacillus* (Winstedt et al. 1998) and the other forming a slightly modified version of the same operon in which the transporter subunits, *cydD* and *cydC*, are transposed in prepended position (figs. 2, 3*B*, and 4*B*). Only the γ-proteobacterium, *S. hydrothermalis*, has the same doublet of gene clusters for *bd* oxidase enzymes, whereas other proteobacteria have a single *cydABDC* cluster (fig. 3). This evidence needs to be qualified with respect to the two different types of bacterial *bd* oxidases that we have characterized in depth, the *bd*-I type and the CIO type. For the latter type, only *Acidocella*, *A. angustum, Defluviimonas,* and a handful of γ-proteobacteria have the *cydABDC* operon of Firmicutes. In contrast, other *Acidiphilium* organisms have CIO oxidases of subtype C, similarly to acetic acid bacteria (figs. 4 and 6), with a different gene cluster which is fundamentally related to the *ythABC* operon of Firmicutes.

In evolutionary terms, this observation creates a puzzle: How did *Acidocella* obtain a set of genes for its CIO *bd* oxidase that is completely different from other acetic acid bacteria (cf. Chouaia et al. 2014), while having the same set of genes for its *bd* oxidase of *bd*-I type? The simplest explanation is that *Acidocella* has retained both duplicate sets derived from an early genomic duplication, which subsequently led to the differentiation of *bd*-I type oxidases. Concomitantly it did not inherit, or maintained only transiently, the CIO type of *bd* oxidases that is present in acetic acid bacteria. In this view, *Acidocella* would constitute a unique crossroad in the molecular evolution of *bd* oxidases: It could be among the progenitors of the *bd*-I type that then diffused to other proteobacteria, maintaining the ancestral form of proteobacterial *bd* oxidases of CIO type, which are classified under subtype A (fig. 4). Such a unique evolutionary situation for *Acidocella* is not restricted to *bd* oxidases, as it applies also to ISP proteins (fig. 5*B*), as well as cytochrome *b*, key subunits of complex I and assimilatory nitrate reductases (Servín-Garcidueñas L, unpublished data). Hence, several bioenergetic systems of α-proteobacteria (Degli Esposti et al. 2014) may have their earliest divergent form in *Acidocella*. This is only in apparent contradiction with the evidence that *Acidocella*, like other acetic acid bacteria, does not usually lie in basal branches of the phylogenomic trees for α-proteobacteria (fig. 5*A*, cf. Williams et al. 2007; Segata et al. 2013; Amrine et al. 2014). These trees are based upon concatenated conserved proteins that do not include the abovementioned proteins involved in membrane redox function and bioenergy production, which have an uneven and complex distribution among bacterial taxa (Degli Esposti et al. 2014) and consequently are considered unsuitable for phylogenomic analysis (Segata et al. 2013).

To conclude, we present here a new classification of the genomic organization of *bd* oxidases that could bear relevance to the phylogenesis of proteobacteria. In particular, we found that the γ-proteobacterium, *S. hydrothermalis*, possesses the same doublet of *cydABDC* operon for *bd* oxidases as the α-proteobacteria, *Acidocella* and *A. angustum*. Although *Salinisphaera* are a moderately halophilic organisms living in marine habitats (Crespo-Medina et al. 2009; Zhang et al. 2012), *Acidocella* lives in strongly acidic environments of wetlands (Dedysh 2011) and lakes (Servín-Garcidueñas et al. 2013). Therefore, an eventual transfer of the *bd* oxidase operon from *Acidocella* to *Salinisphaera* must have occurred a long time ago, when both organisms shared common marine environments, or ancestors. Because *S. hydrothermalis* lies at the root of γ-proteobacteria (supplementary fig. S2, Supplementary Material online; cf. Zhang et al. 2012), its phylogenetic position may be close to the bifurcation in the ancestral α-proteobacterial lineage from which *Acidocella* emerged. Hence, by studying the molecular evolution of *bd* oxidases we have unveiled a possible phylogenetic node in the early separation of the γ- and α-proteobacterial lineages that has in *Acidocella* a potential “living fossil.”

## Supplementary Material

Supplementary table S1 and figures S1 and S2 are available at *Genome Biology and Evolution* online (http://www.gbe.oxfordjournals.org/).

Supplementary Data
