# Supplementary material for: Molecular Evolution of Cytochrome bd Oxidases across Proteobacterial Genomes
Source: Genome Biol Evol. 2015 Feb 16;7(3):801–20. doi: 10.1093/gbe/evv032 (PMC5322542; doi:10.1093/gbe/evv032)
Supplement: Supplementary Data [file supp_7_3_801__index.html]

Molecular evolution of cytochrome bd oxidases across proteobacterial genomes — Molecular Evolution of Cytochrome bd Oxidases across Proteobacterial Genomes — Supplementary Data 

# Molecular Evolution of Cytochrome *bd* Oxidases across Proteobacterial Genomes

## Supplementary Data

files

**Files in this Data Supplement:**

- Supplementary Data - pdf file
- Supplementary Data - docx file
