# Supplementary material for: Molecular Evolution of Cytochrome bd Oxidases across Proteobacterial Genomes
Source: Genome Biol Evol. 2015 Feb 16;7(3):801–20. doi: 10.1093/gbe/evv032 (PMC5322542; doi:10.1093/gbe/evv032)
Supplement: Supplementary Data [file supp_evv032_supplementarymaterialMDEetalrevised.pdf]

Supplementary material

Fig. S1. Simplified view of the distance tree of DeltaBLAST results with 5000 *bd* oxidases.

The NJ tree was obtained with all the results of a broad DELTABLAST search using *cydA* of *Bacillus subtilis* (accession: NP_391755) as a query against 5000 species of proteobacteria excluding most δ-proteobacteria and all ε-proteobacteria, as well as Enterobacterales, to reduce sequence redundancy and the complex distribution of δ-proteobacterial oxidases (see Fig. 1 and main text). Major possible instances of Lateral Gene Transfer (LGT) are indicated (see main text).

Fig. S2. Phylogenomic profile of gamma proteobacteria.

The genomes of a selection of taxa representing all the major orders and families of γ-proteobacteria (cf. Rao and Gupta, 2007; Segata et al, 2013), including the taxa shown in Figs. 3-6 of the main article (shown in bold characters), were analysed and presented in a comprehensive phylogenetic tree as described in the Methods (main text).

Table S1. Compilation of the *bd* oxidases most studied in this work.

Fig. S1 - Simplified view of the distance tree (NJ) of DeltaBLAST 5000 of all cytochrome *bd* oxidases.

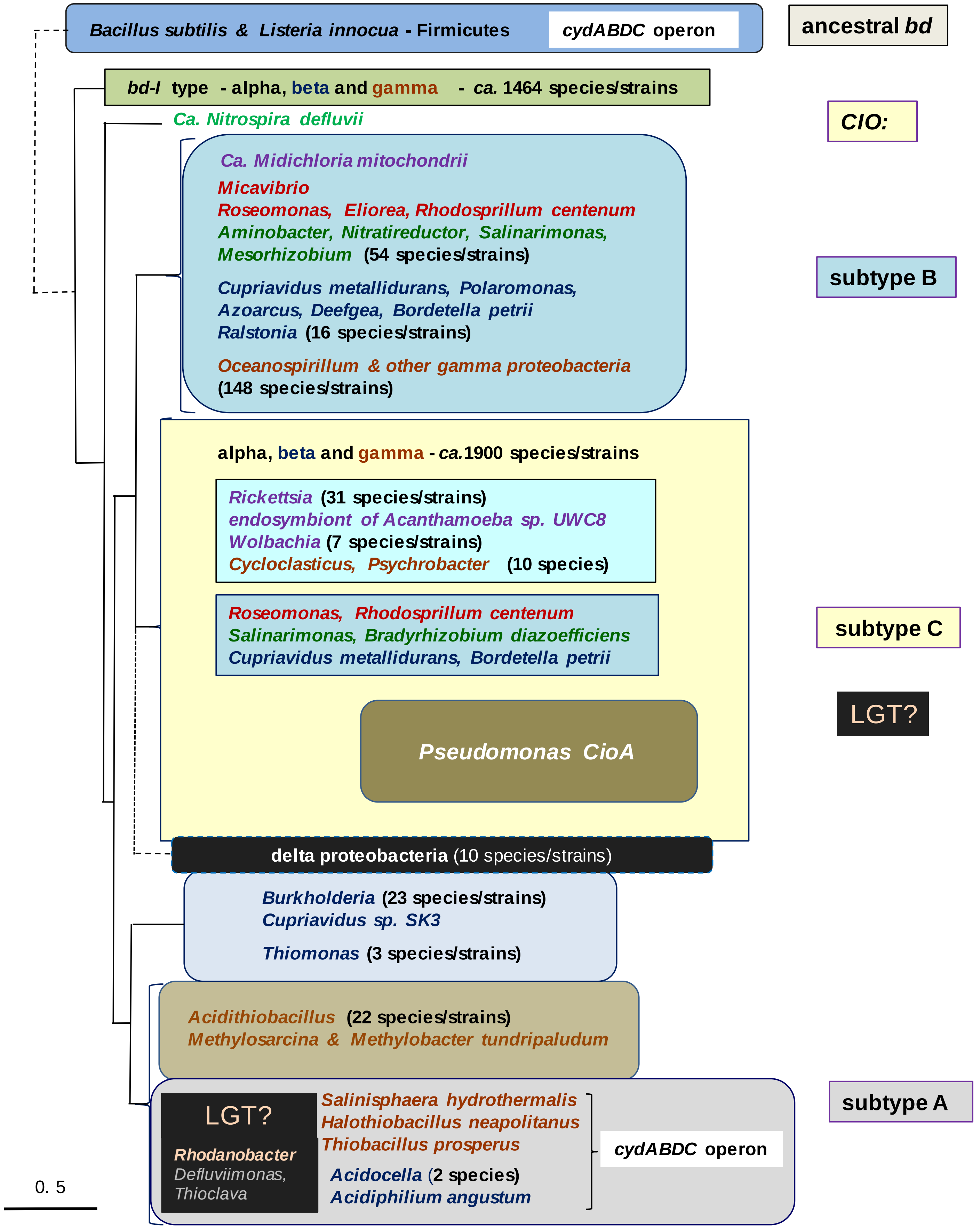

Fig. S1

Figure S2 - Phylogenomics of gamma proteobacteria. In bold are the taxa used in the trees of Figs. 3-6.

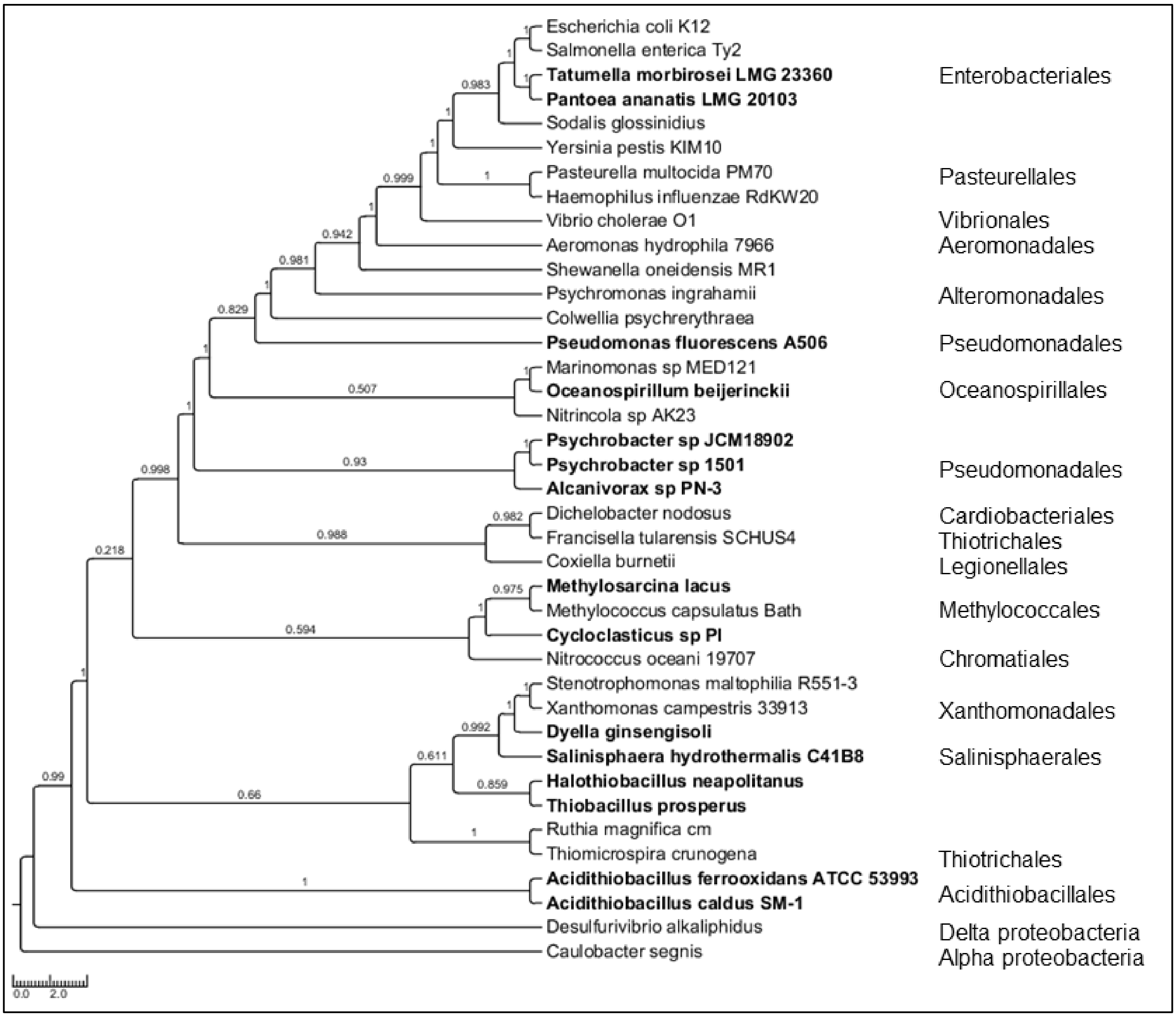

Fig. S2

Table S1. Compilation of the *bd* oxidases most studied in this work.

			cydA (aa)	*cydB* (aa)	
organism	accession	taxonomic group	subunit I	subunit II	classification and notes
		Eubacteria			
Acidocella sp. MX-AZ02	WP_008494833	alpha proteobacteria	480	347	CIO subtype A with *cydABDC* cluster
Acidocella facilis	WP_026438987	alpha proteobacteria	480	347	CIO subtype A with *cydABDC* cluster
Acidiphilium angustum	WP_029312988	alpha proteobacteria	480	348	CIO subtype A with *cydABDC* cluster
Salinisphaera hydrothermalis	KEZ78450	gamma proteobacteria	478	350	CIO subtype A with *cydABDC* cluster
Halothiobacillus neapolitanus	ACX96129	gamma proteobacteria	483	348	CIO subtype A with *cydABDC* cluster
Thiobacillus prosperus	KFZ91409	gamma proteobacteria	480	345	CIO subtype A with *cydABDC* cluster
Tatumella morbirosei	KGD72389	gamma proteobacteria - enterobacteria	481	348	CIO subtype A, *cydDC* prepended
Defluviimonas sp. 20V17	KDB01967	alpha proteobacteria	479	348	CIO subtype A, *cydD* only
Acidithiobacillus caldus	WP_014002943	gamma proteobacteria	484	missing	CIO subtype A degenerate
Rhodanobacter sp. 115	EIL96202	gamma proteobacteria	478	missing	CIO subtype A degenerate
Methylosarcina lacus	WP_029646396	gamma proteobacteria	452	334 in separate cluster	CIO subtype A degenerate
Methylobacter tundripaludum	WP_031436940	gamma proteobacteria	486	336 in separate cluster	CIO subtype A degenerate
Acidithiobacillus thiooxidans	WP_024895144	gamma proteobacteria	482	345	subunit I clustering with subtype A, no *cydDC*
Thiomonas intermedia	Tint_0239, YP_003641981	beta proteobacteria	494	341	subunit I clustering with subtype A, no *cydDC*
Burkholderia sp. BT03	EUC12370	beta proteobacteria	463	328	subunit I clustering with subtype A, no *cydDC*
Ca. Midichloria mitochondrii	midi_00095, YP_004679103	alpha proteobacteria	443	316	CIO subtype B
Rhodospirillum centenum SW	RC1_0287, YP_002296549	alpha proteobacteria	448	335	CIO subtype B
Micavibrio aeruginosavorus	YP_004864645, MICA_298	alpha proteobacteria	457	335	CIO subtype B
Mesorhizobium loti	WP_027062122	alpha proteobacteria	453	334	CIO subtype B
Roseomonas cervicalis ATCC 49957	EFH12552	alpha proteobacteria	447	337	CIO subtype B
Elioraea tepidiphila	WP_019014234	alpha proteobacteria	444	337	CIO subtype B
Terasakiella pusilla	WP_028880378	alpha proteobacteria	449	336	CIO subtype B
Salinarimonas rosea	WP_029030014	alpha proteobacteria	464	340	CIO subtype B
Oceanospirillum beijerinckii	WP_028301376	gamma proteobacteria	453	332	CIO subtype B
Teredinibacter turnerae T7901	TERTU_0322, YP_003071988	gamma proteobacteria	454	334	CIO subtype B
Cupriavidus metallidurans CH34	Rmet_5232 YP_587360	beta proteobacteria	453	345	CIO subtype B
Polaromonas naphthalenivorans CJ2	Pnap_0495 YP_980736	beta proteobacteria	470	343	CIO subtype B
beta proteobacterium L13	WP_017509874	beta proteobacteria	442	336	CIO subtype B
Deefgea rivuli	WP_027467314	beta proteobacteria	445	341	CIO subtype B
Acetobacter pasteurianus 386B	APA386B_1011	alpha proteobacteria	477	338	CIO subtype C
Acetobacter pasteurianus IFO 3283-01	APA01_21500	alpha proteobacteria	477	336	CIO subtype C
Acidiphilium angustum	WP_029312988	alpha proteobacteria	480	348	CIO subtype C
Acidiphilium cryptum	Acry_2040	alpha proteobacteria	472	341	CIO subtype C
Acidiphilium multivorum	ACMV_22850	alpha proteobacteria	472	341	CIO subtype C
Acidomonas methanolica NBRC	GAJ28377	alpha proteobacteria	477	339	CIO subtype C
Agrobacterium radiobacter K84	Arad_12230	alpha proteobacteria	475	335	CIO subtype C
alpha bacterium BAL-199	EDP63365	alpha proteobacteria	473	343	CIO subtype C
Asaia sp. SF2.1	WP_023978129	alpha proteobacteria	469	340	CIO subtype C
Asticcacaulis benevestitus	WP_018084088	alpha proteobacteria	471	337	CIO subtype C
Azorhizobium caulinodans ORS 571	AZC_3759	alpha proteobacteria	478	335	CIO subtype C
Azorhizobium caulinodans ORS 571	AZC_1354	alpha proteobacteria	470	329	CIO subtype C
Azospirillum lipoferum	AZOLI_p30515	alpha proteobacteria	465	339	CIO subtype C
Bartonella tamiae	EJF93580	alpha proteobacteria	468	335	CIO subtype C
Beijerinckia indica	Bind_3288	alpha proteobacteria	462	339	CIO subtype C
Bradyrhizobium diazefficiens USDA	bll0283 &	alpha proteobacteria	468	328	CIO subtype C
Bradyrhizobium oligotrophicum S58	S58_06340	alpha proteobacteria	468	336	CIO subtype C
Bradyrhizobium sp. STM 3843	WP_008973060	alpha proteobacteria	467	343	CIO subtype C
Bradyrhizobium sp. DFCI-1	WP_021079049	alpha proteobacteria	468	333	CIO subtype C
Bradyrhizobium sp. Ec3.3	WP_027526639	alpha proteobacteria	466	336	CIO subtype C
Bradyrhizobium sp. S23321	S23_13360	alpha proteobacteria	469	328	CIO subtype C
endosymbiont of Acanthamoeba sp.	AIF81514	alpha proteobacteria	465	337	CIO subtype C
Geminicoccus roseus	WP_027132750	alpha proteobacteria	479	333	CIO subtype C
Gluconacetobacter or	WP_019086429	alpha proteobacteria	477	339	CIO subtype C
Gluconacetobacter diazotrophicus PAl 5	Gdia_1472	alpha proteobacteria	466	335	CIO subtype C
Gluconacetobacter xylinus NBRC 3288	GLX_30020	alpha proteobacteria	477	339	CIO subtype C
Gluconobacter frateurii	WP_010501967	alpha proteobacteria	474	341	CIO subtype C
Gluconobacter morbifer	EHH67656	alpha proteobacteria	476	341	CIO subtype C
Gluconobacter oxydans 621H	GOX0278	alpha proteobacteria	471	341	CIO subtype C
Gluconobacter oxydans H24	B932_2110	alpha proteobacteria	474	341	CIO subtype C
Granulibacter bethediensis	GbCGDNIH1_013	alpha proteobacteria	482	342	CIO subtype C
Hyphomicrobium sp. MC1	HYPMC_4893	alpha proteobacteria	475	337	CIO subtype C
Inquilinus limosus	WP_026872492	alpha proteobacteria	465	335	CIO subtype C

Table S1… continued

organism	accession	taxonomic group	*cydA* (aa) subunit I	*cydB* (aa) subunit II	classification and notes
		Eubacteria			
**Methylobacterium extorquens DSM**	EHP90754	alpha proteobacteria	**472**	**335**	CIO subtype C
**Methylobacterium nodulans**	Mnod_3333	alpha proteobacteria	**478**	**335**	CIO subtype C
**Methylobacterium populi**	Mpop_5132	alpha proteobacteria	**474**	**336**	CIO subtype C
**Methylobacterium radiotolerans**	Mrad2831_4861	alpha proteobacteria	**470**	**328**	CIO subtype C
**Methylobacterium radiotolerans**	Mrad2831_4927	alpha proteobacteria	**472**	**335**	CIO subtype C
**Methylobacterium sp. 4-46**	M446_4323	alpha proteobacteria	**478**	**334**	CIO subtype C
**Methylobacterium sp. 4-46**	M446_1250	alpha proteobacteria	**482**	**347**	CIO subtype C
**Methylobacterium sp. GXF4**	EIZ81581	alpha proteobacteria	**472**	**335**	CIO subtype C
**Methylobacterium extorquens AM1**	MexAM1_META1 p4206	alpha proteobacteria	**474**	**336**	CIO subtype C
**Methylobacterium extorquens AM1**	MexAM1_META2 p1123 - plasmid	alpha proteobacteria	**472**	**335**	CIO subtype C
**Methylobacterium extorquens DM4**	METDI4817	alpha proteobacteria	**474**	**336**	CIO subtype C
**Methylobacterium extorquens PA1**	Mext_3834	alpha proteobacteria	**474**	**336**	CIO subtype C
**Methylocella silvestris BL2**	Msil_2188	alpha proteobacteria	**472**	**335**	CIO subtype C
**Methylocella silvestris BL2**	Msil_2644	alpha proteobacteria	**474**	**335**	CIO subtype C
**Microvirga lotononidis**	WP_009493026	alpha proteobacteria	**473**	**335**	CIO subtype C
**Oligotropha carboxidovorans OM5**	OCAR_4639	alpha proteobacteria	**468**	**337**	CIO subtype C
**Paracoccus denitrificans PD1222**	Pden_4010	alpha proteobacteria	**467**	**334**	CIO subtype C, also called *qxtAB*
**Parvibaculum lavamentivorans DS-1**	Plav_0596	alpha proteobacteria	**463**	**336**	CIO subtype C
**Phaeobacter gallaeciensis DSM 17395**	PGA1_c19670	alpha proteobacteria	**474**	**334**	CIO subtype C
**Pseudovibrio sp. FO-BEG1**	PSE_1416	alpha proteobacteria	**404**	**334**	CIO subtype C
**Rhizobium sp. CF122**	EJL50761	alpha proteobacteria	**468**	**331**	CIO subtype C
**Rhodobacter sphaeroides 2.4.1**	RSP_3212	alpha proteobacteria	**465**	**335**	CIO subtype C, also called *qxtAB*
**Rhodomichrobium vaniellii**	Rvan_0181	alpha proteobacteria	**464**	**337**	CIO subtype C
**Rhodopseudomonas palustris BisA53**	RPE_0539	alpha proteobacteria	**468**	**338**	CIO subtype C
**Rhodopseudomonas palustris BisB5**	RPD_4378	alpha proteobacteria	**468**	**334**	CIO subtype C
**Rhodopseudomonas palustris CA009**	RPA4793	alpha proteobacteria	**468**	**336**	CIO subtype C
**Rhodopseudomonas palustris TIE-1**	Rpal_1533	alpha proteobacteria	**465**	**341**	CIO subtype C
**Rhodospirillum centenum**	RC1_0416	alpha proteobacteria	**479**	**339**	CIO subtype C
**Rickettsia canadensis str. McKiel**	A1E_01235	alpha proteobacteria	**454**	**339**	CIO subtype C
**Rickettsia conorii str. Malish 7**	RC0288, NP_359925	alpha proteobacteria	**456**	**339**	CIO subtype C
**Rickettsia felis URRWXCal2**	RF_0333	alpha proteobacteria	**456**	**339**	CIO subtype C
**Rickettsia massiliae MTU5**	RMA_0296, YP_001499104	alpha proteobacteria	**430**	**339**	CIO subtype C
**Rickettsia parkeri str. Portsmouth**	MC1_01625, YP_005392544	alpha proteobacteria	**456**	**339**	CIO subtype C
**Rickettsia peacockii strain Rustic**	RPR_01820	alpha proteobacteria	**456**	**339**	CIO subtype C
**Rickettsia typhi str. TH1527**	RTTH1527_01005	alpha proteobacteria	**447**	**339**	CIO subtype C - no COX in genome
**Rickettsiaceae bacterium Os18**	WP_019231091	alpha proteobacteria	**470**	**339**	CIO subtype C
**Roseomonas cervicalis ATCC 49957**	WP_007003071	alpha proteobacteria	**469**	**354**	CIO subtype C
**Salinarimonas rosea**	WP_029032601	alpha proteobacteria	**481**	**347**	CIO subtype C
**Sphingobium xenophagum**	WP_019054127	alpha proteobacteria	**467**	**332**	CIO subtype C
**Starkeya novella**	Snov_0620	alpha proteobacteria	**469**	**340**	CIO subtype C
**Starkeya novella**	Snov_3535	alpha proteobacteria	**474**	**346**	CIO subtype C
**Thalassospira profundimaris**	EKF07979	alpha proteobacteria	**462**	**332**	CIO subtype C
**Tistrella mobilis**	TMO_b0108	alpha proteobacteria	**491**	**336**	CIO subtype C
**Wolbachia endosymbiont of Drosophila melanogaster**	WD0740	alpha proteobacteria	**468**	**340**	CIO subtype C
**Wolbachia wRi**	YP_002727277	alpha proteobacteria	**468**	**340**	CIO subtype C
**Zymomonas mobilis subsp. mobilis NCIMB 11163**	Za10_1638	alpha proteobacteria	**476**	**340**	CIO subtype C
**Pseudomonas fluorescens A506**	PflA506_4640	gamma proteobacteria	**479**	**335**	CIO subtype C, also called *cioAB*
**Cycloclasticus sp. P1**	YP_006836894, Q91_0351	gamma proteobacteria	**470**	**339**	CIO subtype C
**Cycloclasticus pugetii**	WP_020161758	gamma proteobacteria	**470**	**339**	CIO subtype C
**Psychrobacter sp. G**	PSYCG_08730, YP_008163613	gamma proteobacteria	**497**	**379**	CIO subtype C
**Psychrobacter sp. JCM 18902**	GAF58390	gamma proteobacteria	**498**	**338**	CIO subtype C
**Psychrobacter sp. 1501(2011)**	WP_007395587	gamma proteobacteria	**497**	**338**	CIO subtype C
**Azotobacter vinelandii DJ**	Avin_11050	gamma proteobacteria	**476**	**335**	CIO subtype C
**Legionella pneumophila subsp. pneumophila**	LPO_0235	gamma proteobacteria	**456**	**329**	CIO subtype C
**Frateuria aurantia DSM 6220**	Fraau_2907, YP_005378918	gamma proteobacteria	**465**	**335**	CIO subtype C
**Salmonella enterica subsp. enterica serovar Thompson str. RM6836**	IA1_01940 YP_008612210	gamma proteobacteria - **enterobacteria**	**467**	**336**	CIO subtype C
**Burkholderia gladioli**	WP_013690153	beta proteobacteria	**465**	**336**	CIO subtype C
**Ralstonia eutropha JMP134**	Reut_B4101	beta proteobacteria	**483**	**335**	CIO subtype C
**Desulfovibrio magneticus RS-1**	DMR_06960, YP_002952073	delta proteobacteria	**461**	**338**	CIO subtype C
**Desulfococcus multivorans**	WP_020877073	delta proteobacteria	**459**	**332**	CIO subtype C
***Ca.* Nitrospira defluvii**	YP_003798241	Nitrospirales	**447**	**340**	**CIO ancestral**

Table S1… continued

organism	accession	taxonomic group	*cydA* (aa) subunit I	*cydB* (aa) subunit II	classification and notes
		Eubacteria			
Rhodopseudomonas palustris HaA2	RPB_0929, YP_484550	alpha proteobacteria	524	383	*bd* -I, *cydDC* appended
Rhodopseudomonas palustris DX-1	Rpdx1_2434	alpha proteobacteria	528	383	*bd* -I, *cydDC* appended
Pleomorphomonas oryzae	WP_026791401	alpha proteobacteria	527	383	*bd* -I, *cydDC* appended
Rhodospirillum photometricum	RSPPHO_01249	alpha proteobacteria	561	384	*bd* -I, *cydDC* appended
Rhodovulum sp. PH10	WP_008390734	alpha proteobacteria	531	383	*bd* -I, *cydDC* appended
Thioclava sp. DT23-4	KEO53817	alpha proteobacteria	541	382	*bd* -I, *cydDC* appended
Phaeospirillum molischianum	CCG40832	alpha proteobacteria	525	385	*bd* -I, *cydDC* appended
Thioflavicoccus mobilis	Thimo_0355, YP_007242856	gamma proteobacteria	519	377	*bd* -I, *cydDC* appended
Dyella ginsengisoli	WP_017460853	gamma proteobacteria	534	377	*bd* -I, *cydDC* appended
Frateuria aurantia DSM 6220	Fraau_1563, YP_005377664	gamma proteobacteria	528	378	*bd* -I, *cydDC* appended
Pantoea sp. At-9b	Pat9b_04265 YP_004114307	gamma proteobacteria - enterobacteria	480	348	*bd* -I, *cydDC* appended
Pantoea sp. AS-PWVM4	ERK15167	gamma proteobacteria - enterobacteria	480	348	*bd* -I, *cydDC* appended
Tatumella morbirosei enterobacteria	KGD72389	gamma proteobacteria - enterobacteria	481	348	*bd* -I, *cydDC* appended
Kinetoplastibacterium blastocrithidii	BCUE_0079, YP_007449673	beta proteobacteria	521	384	*bd* -I, *cydDC* appended
Desulfurivibrio alkaliphilus AHT2	DaAHT2_0790,	delta proteobacteria	442	342	*bd* -I, *cydDC* appended
Xanthomonadaceae bacterium 3.5X	KGI78673	gamma proteobacteria	522	377	*bd* -I, *cydD* only appended
Coxiella burnetii RSA 493 - cydA2	NP_819973	gamma proteobacteria	521	377	*subunit I clustering with* ***bd*** *-I, no cydDC*
Acidocella sp. MX-AZ02	WP_008494805	alpha proteobacteria	536	379	*bd* -I, *cydDC* prepended
Acidiphilium angustum	WP_029312671	alpha proteobacteria	536	379	*bd* -I, *cydDC* prepended
Acidiphilium multivorum AIU301	YP_004283908	alpha proteobacteria	537	379	*bd* -I, *cydDC* prepended
Acetobacter pasteurianus 386B	APA386B_473	alpha proteobacteria	537	379	*bd* -I, *cydDC* prepended
Gluconacetobacter or Komagataeibacter europaeus	WP_019092290	alpha proteobacteria	536	379	*bd* -I, *cydDC* prepended
Acidomonas methanolica NBRC 104435	GAJ28292	alpha proteobacteria	535	383	*bd* -I, *cydDC* prepended
Roseomonas sp. B5	WP_019461856	alpha proteobacteria	522	382	*bd* -I, *cydDC* prepended
Rubritepida flocculans	WP_027285067	alpha proteobacteria	530	384	*bd* -I, *cydDC* prepended
Tistrella mobilis	TMO_a0189	alpha proteobacteria	537	384	*bd* -I, *cydDC* prepended
Rhodospirillum centenum SW	RC1_4036	alpha proteobacteria	523	384	*bd* -I, *cydDC* prepended
Oceanibaculum indicum	WP_008945285	alpha proteobacteria	530	384	*bd* -I, *cydDC* prepended
Caulobacter sp. K31	Caul_0634	alpha proteobacteria	526	381	*bd* -I, *cydDC* prepended
Caulobacter crescentus NA1000	CCNA_00800	alpha proteobacteria	520	383	*bd* -I, *cydDC* prepended
Methylobacterium extorquens PA1	Mext_1360	alpha proteobacteria	529	384	*bd* -I, *cydDC* prepended
Brucella melitensis biovar Abortus 2308	BAB2_0728	alpha proteobacteria	504	384	*bd* -I, *cydDC* prepended
Brucella suis 1330 or melitensis	BMNI_II0476,	alpha proteobacteria	525	384	*bd* -I, *cydDC* prepended
Rhizobium etli CIAT 652	RHECIAT_PC0000175	alpha proteobacteria	526	384	*bd* -I, *cydDC* prepended
Liberibacter crescens BT-1	B488_01440	alpha proteobacteria	504	366	*bd* -I, *cydDC* prepended
Salinisphaera hydrothermalis	KEZ76806	gamma proteobacteria	526	377	*bd* -I, *cydDC* prepended
Psychrobacter sp. PRwf-1	PsycPRwf_0327	gamma proteobacteria	532	338	*bd* -I, *cydDC* prepended
Variovorax paradoxus B4 - beta	VAPA_1c34620	beta proteobacteria	537	384	*bd* -I, *cydDC* prepended
Burkholderia kururiensis	WP_017772623	beta proteobacteria	529	378	*bd* -I, *cydDC* prepended
Streptomyces fulvissimus DSM 40593	YP_007932425	Actinomycetes	515	333	subunit I similar to ***bd*** **-I**
Listeria innocua	WP_003772850	Firmicutes	510	339 in separate cluster	***bd*** **-I degenerate**
Escherichia coli str. K-12 substr. W3110	b0733, NP_415261	gamma proteobacteria - enterobacteria	522	379	*bd*- I with *cydABX* , prototypic *bd-I*
Vibrio cholerae LMA3984-4	VCLMA_B0643 YP_005632513	gamma proteobacteria - enterobacteria	526	378	*bd*- I with *cydABX*
Pantoea agglomerans - γ	EZI36193	gamma proteobacteria - enterobacteria	470	344	*bd*- I with *cydABX*
Pantoea sp. At-9b - γ	Pat9b_3272 YP_004117124	gamma proteobacteria - enterobacteria	471	344	*bd*- I with *cydABX*
Phaeospirillum fulvum	WP_021133524	alpha proteobacteria	525	384	*bd*- I with *cydABX*
Methylocystis sp. SC2	BN69_2246	alpha proteobacteria	524	379	*bd*- I with *cydAB*
Geminicoccus roseus	WP_027133135	alpha proteobacteria	529	379	*bd*- I with *cydABX*
Pleomorphomonas koreensis	WP_026784703	alpha proteobacteria	525	383	*bd*- I with *cydABX*
Inquilinus limosus	WP_026868948	alpha proteobacteria	525	380	*bd*- I with *cydABX*
Novispirillum itersoni	WP_019645898	alpha proteobacteria	521	380	*bd*- I with *cydABX*
Acidithiobac_ferrooxidans	YP_002219519, Lferr_1070	gamma proteobacteria	544	387	*bd*- I with *cydABX*
Acidithiobac_caldus	YP_004749631, Atc_2281	gamma proteobacteria	546	377	*bd*- I with *cydABX*
Thiorhodococcus drewsii	EGV30141	gamma proteobacteria	534	380	*bd*- I with *cydABX*
Methylobacter luteus	WP_027157314	gamma proteobacteria	521	379	*bd*- I with *cydABX*
Methylosarcina fibrata	WP_020563382	gamma proteobacteria	521	379	*bd*- I with *cydABX*
Azotobacter vinelandii DJ	Avin_19890	gamma proteobacteria	537	379	*bd*- I with *cydABX*
Legionella pneumophila 2300/99 Alcoy	lpa_01861	gamma proteobacteria	510	378	*bd*- I with *cydAB*
Methylococcus capsulatus Bath	YP_113575	gamma proteobacteria	525	378	*bd*- I with *cydABX*
Bordetella avium	BAV0991, YP_785514	beta proteobacteria	525	377	*bd*- I with *cydABX*
Thiomonas intermedia (multispecies)	YP_003641759, Tint_0013	beta proteobacteria	545	377	*bd*- I with *cydABX*
Sideroxydans lithotrophicus ES-1	Slit_1151	beta proteobacteria	521	379	*bd*- I with *cydABX*

Table S1… continued – end.

organism	accession	taxonomic group	*cydA* (aa) subunit I	*cydB* (aa) subunit II	classification and notes
		**Eubacteria**			
**Escherichia coli str. K-12 substr. W3110**	Y75_p0949	gamma proteobacteria - **enterobacteria**	**514**	**378**	***bd*- I with *appABX*,** also called ***bd*- II**
**Methylocystis sp. SC2**	BAB2_0728	alpha proteobacteria	**530**	**381**	***bd*- I with *appABX*,** also called ***bd*- II**
**Caenispirillum salinarum**	WP_009542797	alpha proteobacteria	**532**	**386**	***bd*- I with *appABX***
**Defluviimonas sp. 20V17**	KDB05436	alpha proteobacteria	**531**	**382**	***bd*- I with *appABX***
***Ca.* Paracaedibacter acanthamoebae**	AIK95954	alpha proteobacteria	**512**	**386**	***bd*- I with *appABX***
**Commensalibacter intestini**	WP_008854848	alpha proteobacteria	**500**		***bd*- I with *appABX***
**Coxiella burnetii RSA 493 - *cydA* 1**	CBU_0218, NP_819263	gamma proteobacteria	**516**	**387**	***bd*- I with *appABX***
**Francisella philomiragia subsp. philomiragia ATCC 25017**	Fphi_0633, YP_001677353	gamma proteobacteria	**584**	**398**	***bd*- I with *appABX***
**Dechlorosoma suillum PS**	Dsui_1915	beta proteobacteria	**502**	**379**	***bd*- I with *appABX***
**Azoarcus sp. BH72 - β**	azo1232	beta proteobacteria	**522**	**380**	***bd*- I with *appABX***
**Desulfobulbus propionicus DSM 2032**	Despr_0567, WP_015723288.	delta proteobacteria	**512**	**383**	***bd*- I with *appABX***
**Nitratifractor salsuginis DSM 16511**	Nitsa_0601	epsilon proteobacteria	**510**	**376**	***bd*- I with *appABX***
**Leptospirillum ferriphilum YSK**	AIA30100	Nitrospirales	**534**	**379**	***bd*- I with *appABX***
***Ca.* Odyssella thessalonicensis**	WP_010297580	alpha proteobacteria	**507**	missing	***bd*- I** with *appABX* degenerate
**Bacillus subtilis subsp. subtilis str. 168**	BSU38760, NP_391755	Firmicutes	**468**	**338**	***cydABDC* operon**, prototypic
**Listeria innocua ATCC 33091**	EHN60252	Firmicutes	**510**	**340**	***cydABDC* operon**
**Bacillus selenitireducens**	Bsel_2506	Firmicutes	**459**	**337**	***cydABDC* operon**
**Bacillus selenitireducens**	Bsel_0543	Firmicutes	**452**	**339**	***cydABDC* operon**
**Salsuginibacillus kocurii**	WP_018924928	Firmicutes	**447**	**338**	***cydABDC* operon**
**Exiguobacterium sp. AT1b**	EAT1b_2777	Firmicutes	**462**	**343**	***cydABDC* operon**
**Bacillus amyloliquefaciens**	BAMF_3713	Firmicutes	**468**	**338**	*cydCD* transporters in a separate cluster
**Bacillus subtilis subsp. subtilis str. 168**	BSU30710, NP_390949	Firmicutes	**443**	**346**	***ythABC* operon**
**Bacillus subtilis subsp. natto BEST195**	BSNT_09494, YP_005562287	Firmicutes	**443**	**346**	***ythABC* operon**
**Bacillus cellulosilyticus**	Bcell_0256	Firmicutes	**451**	**337**	***ythABC* operon**
**Bacillus subtilis subsp. subtilis str. RO-NN-1**	I33_3129, YP_005558044	Firmicutes	**443**	**346**	***ythABC* operon**
**Bacillus amyloliquefaciens subsp. plantarum**	BANAU_2983	Firmicutes	**445**	**346**	***ythABC* operon**
**Desulfovibrio magneticus RS-1**	YP_002954207	delta proteobacteria	**433**	**342**	close to *cydABDC* of Firmicutes
**Desulfovibrio sp. X2**	EPR44720	delta proteobacteria	**438**	**341**	close to *cydABDC* of Firmicutes
**Geobacter daltonii**	Geob_0961, YP_002536424	delta proteobacteria	**441**	**340**	close to *cydABDC* of Firmicutes
**Myxococcus xanthus DK 1622**	MXAN_6913, YP_635027	delta proteobacteria	**445**	**342**	close to *cydABDC* of Firmicutes
		**Archaea**			
**Archaeoglobus sulfaticallidus PM70-1**	Asulf_00260, YP_007906286	Euryarchaeota	**506**	**388**	*cydAB* only, no Q loop extension
**Methanosarcina barkeri str. Fusaro**	Mbar_A0121, YP_303687	Euryarchaeota	**439**	**347**	*cydAB* only
**Vulcanisaeta moutnovskia 768-2**	VMUT_1510, YP_004245217	Crenarchaeota	**552**	**372**	*cydAB* only, no Q loop extension
**Vulcanisaeta distributa DSM 14429**	Vdis_1320 , YP_003901757	Crenarchaeota	**469**	**424**	*cydAB* only
**Thermoproteus tenax Kra 1**	TTX_0142, YP_004891899	Crenarchaeota	**458**	**422**	*cydAB* only
**Pyrobaculum neutrophilum V24Sta**	Tneu_0322, YP_001793719	Crenarchaeota	**530**	**367**	*cydAB* only, no Q loop extension
***Ca.* Korarchaeum cryptofilum OPF8**	Kcr_1584, YP_001738012	Korarchaeota	**538**	**367**	*cydAB* only, no Q loop extension
