# Supplementary material for: Molecular Evolution of Cytochrome bd Oxidases across Proteobacterial Genomes
Source: Genome Biol Evol. 2015 Feb 16;7(3):801–20. doi: 10.1093/gbe/evv032 (PMC5322542; doi:10.1093/gbe/evv032)
Supplement: Supplementary Data [file supp_evv032_New_Microsoft_Office_Word_Document.docx]

**Supplementary material**

**Fig. S1. Simplified view of the distance tree of DeltaBLAST results with 5000 *bd* oxidases.** The NJ tree was obtained with all the results of a broad DELTABLAST search using *cydA* of *Bacillus subtilis* (accession: NP_391755) as a query against 5000 species of proteobacteria excluding most δ-proteobacteria and all ε-proteobacteria, as well as Enterobacterales, to reduce sequence redundancy and the complex distribution of δ-proteobacterial oxidases (see Fig. 1 and main text). Major instances of LGT are indicated (see main text).

**Fig. S2. Phylogenomic profile of gamma proteobacteria.** The genomes of a selection of taxa representing all the major orders and families of γ-proteobacteria (cf. Rao and Gupta, 2007; Segata et al, 2013), including the taxa shown in Figs. 2-6 of the main article (shown in bold characters), were analysed and presented in a comprehensive phylogenetic tree as described in the Methods (man text).

**Table S1. Compilation of the *bd* oxidases most studied in this work.**
